# WHO/ILO work-related burden of disease and injury: Protocol for systematic reviews of exposure to occupational ergonomic risk factors and of the effect of exposure to occupational ergonomic risk factors on osteoarthritis of hip or knee and selected other musculoskeletal diseases

**DOI:** 10.1016/j.envint.2018.09.053

**Published:** 2019-04

**Authors:** Carel T.J. Hulshof, Claudio Colosio, Joost G. Daams, Ivan D. Ivanov, K.C. Prakash, Paul P.F.M. Kuijer, Nancy Leppink, Stefan Mandic-Rajcevic, Frederica Masci, Henk F. van der Molen, Subas Neupane, Clas-Håkan Nygård, Jodi Oakman, Frank Pega, Karin Proper, Annette M. Prüss-Üstün, Yuka Ujita, Monique H.W. Frings-Dresen

**Affiliations:** a Coronel Institute of Occupational Health, Amsterdam UMC, location AMC, Amsterdam Public Health Research Institute, Amsterdam, Netherlands; b Department of Health Sciences, University of Milan, Milan, Italy; c International Centre for Rural Heath, University Hospital San Paolo, Milan, Italy; d Department of Public Health, Environmental and Social Determinants of Health, World Health Organization, Geneva, Switzerland; e Faculty of Social Science (Health Sciences), University of Tampere, Tampere, Finland; f Labour Administration, Labour Inspection and Occupational Safety and Health Branch, International Labour Organization, Geneva, Switzerland; g LaTrobe University, Melbourne, Australia; h Centre for Nutrition, Prevention and Health Services, National Institute for Public Health and the Environment, Amsterdam, Netherlands

## Abstract

**Background:**

The World Health Organization (WHO) and the International Labour Organization (ILO) are developing a joint methodology for estimating the national and global work-related burden of disease and injury (WHO/ILO joint methodology), with contributions from a large network of experts. In this paper, we present the protocol for two systematic reviews of parameters for estimating the number of disability-adjusted life years from osteoarthritis of hip or knee, and selected other musculoskeletal diseases respectively, attributable to exposure to occupational ergonomic risk factors to inform the development of the WHO/ILO joint methodology.

**Objectives:**

We aim to systematically review studies on exposure to occupational ergonomic risk factors (Systematic Review 1) and systematically review and meta-analyze estimates of the effect of exposure to occupational ergonomic risk factors on osteoarthritis of the hip or knee, and selected other musculoskeletal diseases respectively (Systematic Review 2), applying the Navigation Guide systematic review methodology as an organizing framework, conducting both systematic reviews in tandem and in a harmonized way.

**Data sources:**

Separately for Systematic Reviews 1 and 2, we will search electronic academic databases for potentially relevant records from published and unpublished studies, including Medline, EMBASE, Web of Science and CISDOC. We will also search electronic grey literature databases, Internet search engines and organizational websites; hand-search reference lists of previous systematic reviews and included study records; and consult additional experts.

**Study eligibility and criteria:**

We will include working-age (≥15 years) workers in the formal and informal economy in any WHO and/or ILO Member State, but exclude children (<15 years) and unpaid domestic workers. The included occupational ergonomic risk factors will be any exposure to one or more of: force exertion; demanding posture; repetitiveness; hand-arm vibration; lifting; kneeling and/or squatting; and climbing. Included outcomes will be (i) osteoarthritis and (ii) other musculoskeletal diseases (i.e., one or more of: rotator cuff syndrome; bicipital tendinitis; calcific tendinitis; shoulder impingement; bursitis shoulder; epicondylitis medialis; epicondylitis lateralis; bursitis elbow; bursitis hip; chondromalacia patellae; meniscus disorders; and/or bursitis knee). For Systematic Review 1, we will include quantitative prevalence studies of any exposure to occupational ergonomic risk factors stratified by country, gender, age and industrial sector or occupation. For Systematic Review 2, we will include randomized controlled trials, cohort studies, case-control-studies and other non-randomized intervention studies with an estimate of the relative effect of any exposure with occupational ergonomic risk factors on the prevalence or incidence of osteoarthritis and/or selected musculoskeletal diseases, compared with the theoretical minimum risk exposure level (i.e., no exposure).

**Study appraisal and synthesis methods:**

At least two review authors will independently screen titles and abstracts against the eligibility criteria at a first stage and full texts of potentially eligible records at a second stage, followed by extraction of data from qualifying studies. At least two review authors will assess risk of bias and the quality of evidence, using the most suited tools currently available. For Systematic Review 2, if feasible, we will combine relative risks using meta-analysis. We will report results using the guidelines for accurate and transparent health estimates reporting (GATHER) for Systematic Review 1 and the preferred reporting items for systematic reviews and meta-analyses guidelines (PRISMA) for Systematic Review 2.

PROSPERO registration number: CRD42018102631.

## Highlights

• WHO and ILO are developing a joint methodology for estimating the national and global work-related burden of disease and injury.
• This article presents a protocol for two systematic reviews for the development of this WHO/ILO joint methodology.
• Prevalence studies on exposure to occupational ergonomic risk factors will be systematically reviewed.
• Studies on effects of occupational ergonomic risk factor exposure on selected musculoskeletal diseases will be systematically reviewed and meta-analysed.

## 1 Background

The World Health Organization (WHO) and the International Labour Organization (ILO) are developing a joint methodology for estimating the work-related burden of disease and injury (WHO/ILO joint methodology) (Ryder, 2017). The organizations plan to estimate the numbers of deaths and disability-adjusted life years that are attributable to selected occupational risk factors, in the first place for the year 2015. The WHO/ILO joint methodology will be based on already existing methodologies of WHO and ILO for estimating the burden of disease for selected occupational risk factors (Prüss-Ustün et al., 2017; International Labour Organization, 2014). It will expand existing methodologies with estimation of the burden of several prioritized additional pairs of occupational risk factors and health outcomes. For this purpose, population attributable fractions (Murray et al., 2004) – the proportional reduction in burden from the health outcome achieved by a reduction of exposure to the risk factor to zero – will be calculated for each additional risk factor-outcome pair, and these fractions will be applied to the total disease burden envelopes for the health outcome from the WHO *Global Health Estimates* (World Health Organization, 2017).

The WHO/ILO joint methodology will likely include the existing and established methodologies for estimating the burdens of low back and neck pain attributable to occupational ergonomic risk factors. In addition, it may however also include a methodology for estimating the burden of osteoarthritis of hip or knee and selected other musculoskeletal diseases (other than low back pain and neck pain) arising from exposure to occupational ergonomic risk factors if feasible, as two additional prioritized risk factor-outcome pairs. Of all musculoskeletal diseases other than low back pain and neck pain, the ones we have selected as “osteoarthritis of hip or knee” and “selected other musculoskeletal diseases” probably accrue the relatively highest occupational disease burdens. To optimize parameters used in estimation models, a systematic review is required of studies on the prevalence of exposure to occupational ergonomic risk factors (‘Systematic Review 1’), as well as a second systematic review and meta-analysis of studies with estimates of the effect of exposure to occupational ergonomic risk factors on osteoarthritis of hip or knee, and selected other musculoskeletal diseases respectively, (‘Systematic Review 2’). In the current paper, we present the protocol for these two systematic reviews, in parallel to presenting systematic review protocols on other additional risk factor-outcome pairs elsewhere (Descatha et al., 2018; Godderis et al., 2018; Li et al., 2018; Mandrioli et al., 2018; Paulo et al., 2018; Rugulies et al., Accepted; Teixeira et al., 2018; Tenkate et al., 2018). To our knowledge, this is the first systematic review protocol of its kind. The WHO/ILO joint estimation methodology and the burden of disease estimates are separate from these systematic reviews, and they will be described and reported elsewhere.

We refer separately to Systematic Reviews 1 and 2, because the two systematic reviews address different objectives and therefore require different methodologies. The two systematic reviews will, however, be harmonized and conducted in tandem. This will ensure that – in the later development of the methodology for estimating the burden of disease from this risk factor-outcome pair – the parameters on the risk factor prevalence are optimally matched with the parameters from studies on the effect of the risk factor on the designated outcome. The findings from Systematic Reviews 1 and 2 will be reported in two distinct journal articles.

### 1.1 Rationale

To consider the feasibility of estimating the burden of selected musculoskeletal diseases from exposure to occupational ergonomic risk factors, and to ensure that potential estimates of burden of disease are reported in adherence with the guidelines for accurate and transparent health estimates reporting (GATHER) (Stevens et al., 2016), WHO and ILO require a systematic review of studies on the prevalence of relevant levels of exposure to occupational ergonomic risk factors (Systematic Review 1), as well as a systematic review and a meta-analysis of studies with estimates of the relative effect of exposure to occupational ergonomic risk factors on the prevalence or incidence of osteoarthritis of hip or knee, and selected other musculoskeletal diseases respectively, compared with the theoretical minimum risk exposure level (Systematic Review 2). The theoretical minimum risk exposure level is the level that would result in the lowest possible population risk, even if it is not feasible to attain this exposure level in practice (Murray et al., 2004). These data and effect estimates should be tailored to serve as parameters for estimating the burden of osteoarthritis of hip or knee, and selected other musculoskeletal diseases respectively, from exposure to occupational ergonomic risk factors in the WHO/ILO joint methodology.

We are not aware of any previous systematic reviews of the existing evidence on the exposure to any of the occupational ergonomic risk factors covered in this review (i.e., one or more of: force exertion, demanding posture, repetitiveness, hand-arm vibration, lifting, kneeling and/or squatting, and climbing) independent of a specific disease (Systematic Review 1). Seven previous systematic reviews have however focused on the evidence on the effect of exposure to one or more of these occupational ergonomic risk factors on one or more selected musculoskeletal diseases of the shoulder (van Rijn et al., 2010; van der Molen et al., 2017; Lievense et al., 2001); elbow (Descatha et al., 2016); hip (Jensen, 2008; Lievense et al., 2001); and knee (Verbeek et al., 2017) (Systematic Review 2). These systematic reviews identified the following occupational ergonomic risk factors as relevant.

Regarding knee osteoarthritis, Verbeek et al. (2017) concluded in a meta-analysis of 12 case control studies that measured exposure to kneeling or squatting resulted in a summary OR of 1.7 (95% CI 1.35–2.13, I^2^ 49%); exposure to lifting (11 studies) in an OR of 1.69 (95% CI 1.43–2.00, I^2^ 51%); exposure to climbing (seven studies) in an OR of 1.6 (95% CI 1.25–1.91, I^2^ 68%) and a combination of kneeling and lifting (one study) in an OR of 1.35 (95% CI 1.05–1.73) (Verbeek et al., 2017).

A recent meta-analysis, based on 7 studies, revealed moderate quality evidence for associations between shoulder disorders (M75.1-M75.5) and arm elevation (odds ratio (OR) 1.9, 95% confidence interval (CI) 1.47 to 2.47, I^2^ 31%) and shoulder load, a combined biomechanical exposure measure (OR = 2.0, 95% CI 1.90 to 2.10, I^2^ 0%) and low to very low evidence for hand force exertion (OR = 1.5, 95% CI 1.25 to 1.87, I^2^ 66%), and hand-arm vibration (OR = 1.3, 95% CI 1.01 to 1.77, I^2^ 99%) (van der Molen et al., 2017). van Rijn et al. (2010) performed a systematic review on the relationship between work-related factors and specific disorders of the shoulder and found in the 17 included studies that repetitive movements of the shoulder, repetitive motion of the hand/wrist of >2 h/day, hand–arm vibration, and arm elevation showed an association with subacromial impingement syndrome (ORs between: 1.04, 95% CI 1.00–1.07 and 4.7, 95% CI 2.07–10.68), as did upper-arm flexion of ≥45° for ≥15% of time (OR 2.43, 95% CI 1.04–5.68) and duty cycle of forceful exertions of ≥9% time or any duty cycle of forceful pinch (OR 2.66, 95% CI 1.26–5.59) (van Rijn et al., 2010).

Descatha et al. (2016) included in a meta-analysis five prospective studies published between 2001 and 2014 and found a positive association between combined biomechanical exposure involving the wrist and/or elbow and incidence of epicondylitis lateralis (OR 2.6, 95% CI 1.9–3.5) (Descatha et al., 2016). In a systematic review by van Rijn et al. (2009) the associations between force, posture, repetitiveness, hand-arm vibration and a mixture of these exposures and elbow disorders were studied (van Rijn et al., 2009). Handling tools of >1 kg (ORs of 2.1–3.0), handling loads of >20 kg at least ten times/day (OR 2.6) and repetitive movements for >2 h/day (ORs of 2.8–4.7) were associated with lateral epicondylitis, while handling loads of >5 kg (2 times/min at minimum of 2 h/day), handling loads of >20 kg for at least ten times/day, high hand grip forces for >1 h/day, repetitive movements for >2 h/day (ORs of 2.2–3.6) and working with vibrating tools for >2 h/day (OR 2.2) were all associated with medial epicondylitis.

Jensen (2008) evaluated the association between physical work demands and hip osteoarthritis in 22 included studies and concluded that moderate to strong evidence exists for a relation with heavy lifting (OR ranges between 1.97, 95% CI 1.14–3.4, and 8.5 (95% CI 1.6–45.3) (Jensen, 2008). Furthermore, thirteen studies showed a significantly increased risk between farming and hip osteoarthritis, with ORs ranging from 1.9 (95% CI 1.01–3.87) to 12.0 (95% CI 6.7–21.4). Lievense et al. (2001) used a best-evidence synthesis to summarize the results of two retrospective and 14 case-control studies and found moderate evidence for a positive association between previous physical workload and hip osteoarthritis, with ORs ranging from 1.5 (95% CI 0.9–2.5) and 9.3 (95% CI 1.9–44.5) (Lievense et al., 2001). In a subgroup analysis, also ≥10 years farming was positively related to hip osteoarthritis.

Work in the informal economy may lead to different exposures and exposure effects than does work in the formal economy. The informal economy is defined as “all economic activities by workers and economic units that are – in law or in practice – not covered or insufficiently covered by formal arrangements”, but excluding “illicit activities, in particular the provision of services or the production, sale, possession or use of goods forbidden by law, including the illicit production and trafficking of drugs, the illicit manufacturing of and trafficking in firearms, trafficking in persons, and money laundering, as defined in the relevant international treaties” (p. 4) (104th International Labour Conference, 2015). Therefore, we consider the formality of the economy studied in studies included in both systematic reviews.

### 1.2 Description of the risk factors

The aforementioned seven systematic reviews on the effect of occupational ergonomic risk factors on musculoskeletal diseases of the shoulder (van Rijn et al., 2010; van der Molen et al., 2017); elbow (Descatha et al., 2016); hip (Jensen, 2008; Lievense et al., 2001); and knee (Verbeek et al., 2017), and additional documents (Harris and Coggon, 2015; European Agency for Safety and Health at Work, 2010) have identified the seven following types of occupational ergonomic risk factors as of interest: (i) force exertion (e.g., carrying or moving heavy loads, turn and screw); (ii) demanding posture (e.g. arm elevation, bending and/or twisting); (iii) repetitiveness (e.g., physically repetitive work); (iv) hand-arm vibration; (v) kneeling and/or squatting; (vi) lifting (e.g. lifting heavy loads); and/or (vii) climbing. We will review studies on occupational exposure to any (i.e., one or more) of these seven different ergonomic risk factors. The definition of the risk factor, the risk factor levels and the theoretical minimum risk exposure level are presented in Table 1. The WHO burden of disease study has previously defined occupational ergonomic risk factors into four categories by occupation, these being background exposure (defined by manager and professionals as occupations); low exposure (clerical and sales workers); moderate exposure (operators and service workers); and high exposure (farmers) (Murray et al., 2004). The Institute of Health Metrics and Evaluation's burden of disease study has defined occupational ergonomic factors for low back and neck pain specifically as “All individuals have the ergonomic factors of clerical and related workers” (p. 1362) (G. B. D. Risk Factors Collaborators, 2017).

**Table 1:** Definitions of the risk factor, risk factor levels and the minimum risk exposure level.

Risk factor	Exposure to occupational ergonomic risk factors (defined as exposure occupationally to one or more of: force exertion, demanding posture, repetitive movement, hand-arm vibration, kneeling or squatting, lifting and/or climbing)
Risk factor level	Two levels: 1. No exposure to occupational ergonomic risk factors. 2. Any exposure to occupational ergonomic risk factors. If possible, “any” exposure may be further classified into “moderate” and “high” exposure, preferably based on exposure in terms of level, frequency and/or duration.
Theoretical minimum risk exposure level	No exposure to occupational ergonomic risk factors

### 1.3 Description of the outcomes

In Systematic Review 2, we will review two outcomes:

1. Osteoarthritis of the hip or knee.
2. Any selected other musculoskeletal diseases, defined as one or more of: shoulder disorders: rotator cuff syndrome, bicipital tendinitis, calcific tendinitis, shoulder impingement, bursitis shoulder; elbow disorders: epicondylitis medialis, epicondylitis lateralis, bursitis elbow; hip disorders: trochanter and other hip bursitis; and knee disorders: chondromalacia patella, meniscus disorders and bursitis knee.

For the outcome “Any other selected musculoskeletal diseases”, only diseases have been included, for which exposure to one or more of the included occupational ergonomic risk factors (Table 1) is considered as a necessary factor for disease development. This selection was mainly based on the information about a possible occupational origin of the selected health outcomes in the seven systematic reviews described above (van der Molen et al., 2017; van Rijn et al., 2010;Descatha et al., 2016; van Rijn et al., 2009; Jensen, 2008; Lievense et al., 2001; Verbeek et al., 2017), plus additional evidence (Harris and Coggon, 2015; European Agency for Safety and Health at Work, 2010).

The WHO *Global Health Estimates* group outcomes into standard burden of disease categories (World Health Organization, 2017), based on standard codes from the *International Statistical Classification of Diseases and Related Health Problems 10th Revision* (ICD-10) (World Health Organization, 2015). The relevant WHO *Global Health Estimates* categories for Systematic Review 2 are “*II.M.2. Osteoarthritis*” and “*II.M.5. Other musculoskeletal diseases*” (World Health Organization, 2017). Table 2 presents the pairs of occupational ergonomic risk factors and the musculoskeletal diseases included in this review. Table 3 presents for each disease or health problem included in the WHO *Global Health Estimates* categories its inclusion in Systematic Review 2. For both categories, this review does not cover all the relevant WHO *Global Health Estimates* categories.

**Table 2:** Pairs of occupational ergonomic risk factors and musculoskeletal diseases included in this review.

Pair	Risk factor	Disease or health problems
1	Exposure to occupational force exertion	M16, M17, M22.4, M23.0, M23.2, M23.3, M23.4, M70.0-M70.7, M75.1-M75.5, M70.0-M70.1
2	Exposure to occupational demanding posture	M22.4, M23.0, M23.2, M23.3, M23.4, M70.0-M70.7, M75.1-M75.5, M70.0-M70.1
3	Exposure to occupational repetitive body movement	M22.4, M23.0, M23.2, M23.3, M23.4, M70.0-M70.7, M75.1-M75.5, M70.0-M70.1
4	Exposure to occupational hand-arm vibration	M70.0-M70.3, M75.1-M75.5
5	Exposure to occupational kneeling and/or squatting	M17, M22.4, M70.4-M70.5
6	Exposure to occupational lifting	M16, M17,
7	Exposure to occupational climbing	M17

**Table 3:** ICD-10 codes and disease and health problems covered by the WHO *Global Health Estimates* categories “*II.M.2. Osteoarthritis*” and “*II.M.5. Other musculoskeletal diseases*” and their inclusion in Systematic Review 2.

ICD-10 code	Disease or health problems (or groups of diseases)	Inclusion in Systematic Review 2
II.M.2. Osteoarthritis		
M15	Polyarthrosis	No
M16	Coxarthrosis [arthrosis of hip]	Yes
M17	Gonarthrosis [arthrosis of knee]	Yes
M18	Arthrosis of first carpometacarpal joint	No
M19	Other arthrosis	No

II.M.5. Other musculoskeletal diseases		
M00	Pyogenic arthritis	No
M02	Reactive arthropathies	No
M08	Juvenile arthritis	No
M11	Other crystal arthropathies	No
M12	Other specific arthropathies	No
M13	Other arthritis	No
M20	Acquired deformities of fingers and toes	No
M21	Other acquired deformities of limbs	No
M22 (except M22.4)	Disorders of patella	No
M22.4	Chondromalacia patellae	Yes
M23 (except M23.0, M23.2, M23.3)	Internal derangement of knee	No
M23.0	Cystic meniscus	Yes
M23.2	Derangement of meniscus due to old tear or injury	Yes
M23.3	Other meniscus derangements	Yes
M23.4	Loose body in knee	Yes
M24	Other specific joint derangements	No
M25	Other joint disorders, not classified	No
M30–36	Systemic connective tissue disorders	No
M40-M43	Deforming dorsopathies	No
M60-M63	Disorders of muscles	No
M70.0 - M70.1	Bursitis & synovitis hand, wrist	Yes
M70.2 - M70.3	Olecranon & other elbow bursitis	Yes
M70.4 - M70.5	Prepatellaris & other knee bursitis	Yes
M70.6 - M70.7	Trochanter & other hip bursitis	Yes
M71-M73	Other bursopathies, fibroblastic disorders, soft tissue disorders in diseases classified elsewhere	No
M75 (except M75.1-M75.5)	Shoulder lesions	No
M75.1	Rotator cuff syndrome	Yes
M75.2	Bicipital tendinitis	Yes
M 75.3	Calcific tendinitis of shoulder	Yes
M75.4	Impingement syndrome of shoulder	Yes
M75.5	Bursitis of shoulder	Yes
M76	Enthesopathies lower limb	No
M77 (except M77.0-M77.1)	Other enthesopathies	No
M77.0	Epicondylitis medialis	Yes
M77.1	Epicondylitis lateralis	Yes
M80–85	Disorders of bone density and structure	No
M86–90	Other osteopathies	No
M91–M94	Chondropathies	No
M95	Other acquired deformities	No
M96	Postprocedural musculoskeletal disorders	No
M99	Biomechanical lesions, not elsewhere	No

### 1.4 How the risk factors may impact the outcome

**Fig. 1:**
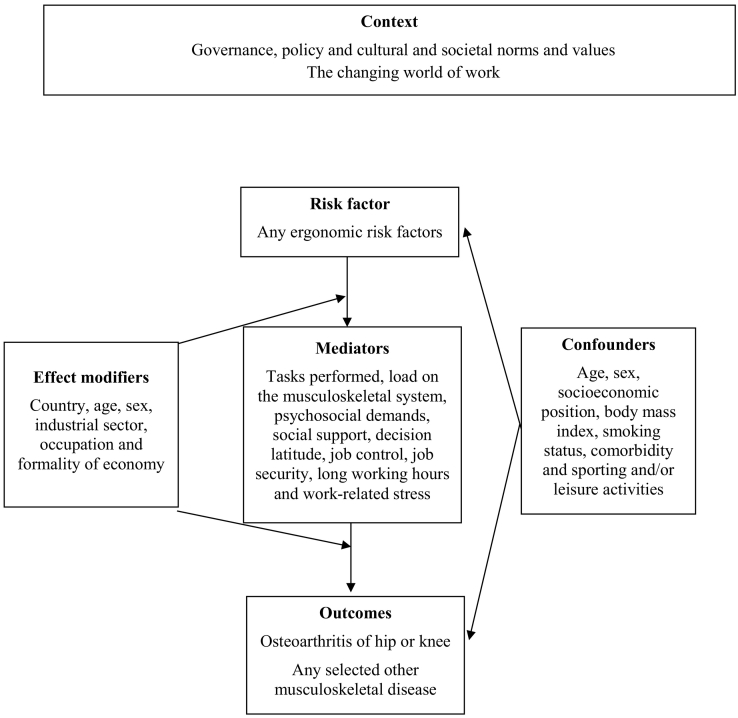
Logic model of the possible causal relationship between exposure to occupational ergonomic risk factors and osteoarthritis of hip or knee and selected other musculoskeletal diseases.

Fig. 1 presents the logic model for our systematic reviews of the causal relationship between exposure to occupational ergonomic risk factors and osteoarthritis, and selected other musculoskeletal diseases respectively. This logic model is an *a priori*, process model (Rehfuess et al., 2018) that seeks to capture complexity of the risk factor-outcome causal relationship (Anderson et al., 2011).

Musculoskeletal diseases are multifactorial in origin, which means that there may be several aetiological risk factors for their onset. Specific potentially relevant pathomechanisms include: posturally induced muscular imbalance, neural pathomechanisms, the ‘Cinderella hypothesis’ of motor unit recruitment, reperfusion, impaired heat-shock response and stress-induced mitochondrial damage (Forde et al., 2002). Nevertheless, there currently is no clear and circumscriptive understanding of the pathogenesis of work-related musculoskeletal diseases. One postulation is that musculoskeletal diseases result from cumulative micro damage induced by risk factors on cellular and/or tissue level over time.

## 2 Objectives

1. Systematic Review 1: To systematically review quantitative studies of any design on the prevalence of relevant levels of exposure to occupational ergonomic risk factors in the years 2005 to 2018 among working-age workers, disaggregated by country, sex, age and industrial sector or occupation.
2. Systematic Review 2: To systematically review and meta-analyze randomized control trials, cohort studies, case-control studies and other non-randomized intervention studies with estimates of the relative effect of any occupational exposure to ergonomic risk factors on osteoarthritis, and selected other musculoskeletal diseases respectively, in any year among working-age workers, compared with the minimum risk exposure level of no exposure.

## 3 Methods

We will apply the *Navigation Guide* (Woodruff and Sutton, 2014) methodology for systematic reviews in environmental and occupational health as our guiding methodological framework, wherever feasible. The guide applies established systematic review methods from clinical medicine, including standard Cochrane Collaboration methods for systematic reviews of interventions, to the field of environmental and occupational health to ensure systematic and rigorous evidence synthesis on environmental and occupational risk factors that reduces bias and maximizes transparency (Woodruff and Sutton, 2014). The need for further methodological development and refinement of the relatively novel *Navigation Guide* has been acknowledged (Woodruff and Sutton, 2014).

Systematic Review 1 may not map well to the *Navigation Guide* framework (Fig. 1 on page 1009 in (Lam et al., 2016c)), which is tailored to hazard identification and risk assessment. Nevertheless, steps 1–6 for the stream on human data can be applied to systematically review exposure to risk factors. Systematic Review 2 maps more closely to the *Navigation Guide* framework, and we will conduct steps 1–6 for the stream on human data, but not conduct any steps for the stream on non-human data, although we will briefly summarize narratively the evidence from non-human data that we are aware of.

We have registered the protocol in PROSPERO under CRD42018102631. This protocol adheres with the preferred reporting items for systematic review and meta-analysis protocols statement (PRISMA-P) (Moher et al., 2015; Shamseer et al., 2015), with the abstract adhering with the reporting items for systematic reviews in journal and conference abstracts (PRISMA-A) (Beller et al., 2013). Any modification of the methods stated in the present protocol will be registered in PROSPERO and reported in the systematic review itself under the section ‘Differences between protocol and review’. Systematic Review 1 will be reported according to the GATHER guidelines (Stevens et al., 2016), and Systematic Review 2 will be reported according to the preferred reporting items for systematic review and meta-analysis statement (PRISMA) (Liberati et al., 2009). Our reporting of all parameters for estimating the burden of osteoarthritis, and other musculoskeletal diseases respectively, from exposure to occupational ergonomic risk factors in the systematic reviews will adhere with the requirements of the GATHER guidelines (Stevens et al., 2016), because the WHO/ILO burden of disease estimates that may be produced consecutive to the systematic review must also adhere to these reporting guidelines.

### 3.1 Systematic Review 1

#### 3.1.1 Eligibility criteria

The population, exposure, comparator and outcome (PECO) criteria (Liberati et al., 2009) are described below.

##### 3.1.1.1 Types of populations

We will include studies of the working-age population (≥15 year) in the formal and informal economy. Studies of children (<15 years) and unpaid domestic workers will be excluded. Participants residing in any WHO and/or ILO Member State and workers in any industrial setting and occupation will be included. Appendix A provides a brief overview of the PECO criteria.

##### 3.1.1.2 Types of exposures

We will include studies that define exposure to occupational ergonomic risk factors in accordance with our standard definition (Table 1). For osteoarthritis of hip or knee, and any selected other musculoskeletal disease respectively, cumulative exposure (e.g. total number of years or total amount of work performed according to one's job history) may be the most biologically relevant exposure metric in theory, but – as done in other burden of disease studies – we will here also prioritize a non-cumulative exposure metric in practice (i.e. exposure in present work calculated solemnly on a daily basis and not taking into account the total exposure history). We believe that insufficient cumulative exposure data currently exist to enable burden of disease estimation.

We will include studies on the prevalence of exposure to the respective occupational risk factor, if it is disaggregated by country (defined as a WHO and/or ILO Member State), sex (two categories: female, male), age (ideally in 5-year age bands, such as 20–24 years) and industrial sector (e.g., *International Standard Industrial Classification of All Economic Activities, Revision 4* [ISIC Rev. 4]) (United Nations, 2008) or occupation (as defined, for example, by the *International Standard Classification of Occupations 1988* [ISCO-88] (International Labour Organization, 1987) or *2008* [ISCO-08] (International Labour Organization, 2012)). Criteria may be revised in order to identify optimal data disaggregation to enable subsequent estimation of the burden of disease.

We shall include studies with exposure data for the years 2005 to 31 July 2018. For optimal modelling of exposure, WHO and ILO require exposure data up to 2018, because recent data points help better estimate time trends, especially where data points may be sparse. The additional rationale for this data collection window is that the WHO and ILO aim to estimate burden of disease in the year 2015, and we believe that the lag time from exposure to outcome will not exceed 10 years (Haibing et al., 2006); so in their models, the organizations can use the exposure data from as early as 2005 to determine the burden of osteoarthritis of hip or knee, and the selected other musculoskeletal diseases respectively, 10 years later in 2015. To make a conclusive judgment on the best lag time to apply in the model, we will summarize the body of evidence on the lag time between exposure to occupational ergonomic risk factors and osteoarthritis of hip or knee, and any selected other musculoskeletal disease respectively in the review.

Both objective and subjective measures will be included. If both subjective and objective measures are presented, then we will prioritize objective ones. Studies with measures from any data source, including registries, will be eligible. The exposure parameter should match the one used in Systematic Review 2 or can be converted to match it.

##### 3.1.1.3 Types of comparators

There will be no comparator, because we will review risk factor prevalence only.

##### 3.1.1.4 Types of outcomes

The outcome is exposure to the occupational risk factor (i.e., ergonomic risk factors).

##### 3.1.1.5 Types of studies

This systematic review will include quantitative studies of any design, including cross-sectional studies. These studies must be representative of the relevant industrial sector, relevant occupational group or the national population. We will exclude qualitative, modelling and case studies, as well as non-original studies without quantitative data (e.g. letters, commentaries and perspectives).

Study records written in any language will be included. If a study record is written in a language other than those spoken by the authors of this review or those of other reviews (Descatha et al., 2018; Godderis et al., 2018; Li et al., 2018; Mandrioli et al., 2018; Paulo et al., 2018; Rugulies et al., Accepted; Teixeira et al., 2018; Tenkate et al., 2018) in the series (i.e. Arabic, Bulgarian, Chinese, Danish, Dutch, English, French, Finnish, German, Hungarian, Italian, Japanese, Norwegian, Portuguese, Russian, Spanish, Swedish and Thai), it will be translated into English. Published and unpublished studies will be included.

Studies conducted using unethical practices will be excluded from the review.

##### 3.1.1.6 Types of effect measures

We will include studies with a measure of the prevalence of exposure to occupational ergonomic risk factors.

#### 3.1.2 Information sources and search

##### 3.1.2.1 Electronic academic databases

We (JGD, HFVM, SM and SN) will at a minimum search the five following electronic academic databases:

1. Ovid Medline with Daily Update (1 January 2005 to 31 July 2018).
2. PubMed (1 January 2005 to 31 July 2018).
3. EMBASE (1 January 2005 to 31 July 2018).
4. Web of Science with inclusion of three databases: Science Citation Index Expanded (1 January 2005 to 31 July 2018); Social Sciences Citation Index (1 January 2005 to 31 July 2018); and Arts and Humanities Citation Index (1 January 2005 to 31 July 2018).
5. OSH UPDATE with inclusion of three databases: CISDOC (1 January 2005 to 31 July 2018); HSELINE (1 January 2005 to 31 July 2018); and NIOSHTIC-2 (1 January 2005 to 31 July 2018).

The Ovid Medline search strategy for Systematic Review 1 is presented in Appendix B. We will perform searches in electronic databases operated in the English language using a search strategy in the English language. Consequently, study records that do not report essential information (i.e. title and abstract) in English will not be captured. We will adapt the search syntax to suit the other electronic academic and grey literature databases. When we are nearing completion of the review, we will search the PubMed database for the most recent publications (e.g., e-publications ahead of print) over the last six months. Any deviation from the planned search strategy in the actual search strategy will be documented.

##### 3.1.2.2 Electronic grey literature databases

JGD, PK, FM and HFVM will at a minimum search the two following electronic grey literature databases:

1. OpenGrey (http://www.opengrey.eu/).
2. Grey Literature Report (http://greylit.org/).

##### 3.1.2.3 Internet search machines

We (JGD, FM, HFVM and SN) will also search the Google (www.google.com/) and Google Scholar (www.google.com/scholar/) Internet search engines and screen the first 100 hits for potentially relevant records, as has been done previously in Cochrane Reviews (Pega et al., 2015; Pega et al., 2017).

##### 3.1.2.4 Organizational websites

At a minimum, the websites of the eight following international organizations and national government departments will be searched by PK, PPMFK and FM:

1. International Labour Organization (www.ilo.org/).
2. World Health Organization (www.who.int).
3. European Agency for Safety and Health at Work (https://osha.europa.eu/en).
4. Eurostat (www.ec.europa.eu/eurostat/web/main/home).
5. China National Knowledge Infrastructure (http://www.cnki.net/).
6. Finnish Institute of Occupational Health (https://www.ttl.fi/en/).
7. United States National Institute of Occupational Safety and Health (NIOSH), using the NIOSH data and statistics gateway (https://www.cdc.gov/niosh/data/).
8. International Ergonomics Association (http://www.iea.cc/).

##### 3.1.2.5 Hand-searching and expert consultation

We (CTJH, PPMFK, FM and SN) will hand-search for potentially eligible studies in:

• Reference lists of previous systematic reviews.
• Reference lists of all study records of all included studies.
• Study records published over the past 24 months in the three peer-reviewed academic journals from which we obtain the largest number of included studies.
• Study records that have cited an included study record (identified in Web of Science citation database).
• Collections of the review authors.

Additional experts will be contacted with a list of included studies and study records, with the request to identify potentially eligible additional ones.

#### 3.1.3 Study selection

Study selection will be carried out with Covidence (Babineau, 2014; *Covidence Systematic Review Software*) or the Rayyan Systematic Reviews Web App (Ouzzani et al., 2016). All study records identified in the search will be downloaded and duplicates will be identified and deleted. Afterwards, two review authors (HFVM and SN) will independently screen titles and abstracts (step 1) and then full texts (step 2) of potentially relevant records. A third review author (PPMFK) will resolve any disagreements between the two review authors. If a study record identified in the literature search was authored by a review author assigned to study selection or if a assigned review author was involved the study that the study record reports, then the record will be re-assigned to another review author for study selection. In the systematic review, we will document the study selection in a flow chart, as per GATHER guidelines (Stevens et al., 2016).

#### 3.1.4 Data extraction and data items

A data extraction form will be developed and piloted until there is convergence and agreement among data extractors. At a minimum, three review authors (FM, HFVM and SN) will independently extract the data on exposure to ergonomic risk factors, disaggregated by country, sex, age and industrial sector or occupation. A fourth and fifth review author (CTJH and CHN) will resolve conflicting extractions, if any. At a minimum, we will extract data on study characteristics (including study authors, study year, study country, participants and exposure), study design (including study type and measurement of occupational exposure with the risk factor), risk of bias (including missing data, as indicated by response rate and other measures) and study context. The estimates of the proportion of the population that is exposed to the occupational risk factor from included studies will be entered into and managed with the Review Manager, Version 5.3 (RevMan 5.3) (*Review Manager (RevMan). Version 5.3,* 2014) or DistillerSR (EvidencePartner, 2017) softwares.

We will also extract data on potential conflict of interest in included studies, including the financial disclosures and funding sources of each author and their affiliated organization. We will use a modification of a previous method to identify and assess undisclosed financial interests (Forsyth et al., 2014). Where no financial disclosure/conflict of interest is provided, we will search declarations of interest both in other records from this study published in the 36 months prior to the included study record and in other publicly available repositories (Drazen et al., 2010a; Drazen et al., 2010b).

If we require any missing data, we will request these from the principal study author by email or phone, using the contact details provided in the principal study record. If we do not receive a response, we will follow up twice via email, at two and four weeks.

#### 3.1.5 Risk of bias assessment

Generally agreed methods (i.e. framework plus tool) for assessing risk of bias do not exist for systematic reviews of input data for health estimates (The GATHER Working Group, 2016), for burden of disease studies, of prevalence studies in general (Munn et al., 2014), and those of prevalence studies of occupational and/or environmental risk factors specifically (Krauth et al., 2013; Mandrioli and Silbergeld, 2016; Vandenberg et al., 2016). None of the five standard risk of bias assessment methods in systematic reviews in environmental and occupational health (Rooney et al., 2016) are applicable to assessing prevalence studies. The *Navigation Guide* does not support checklist approaches, such as (Hoy et al., 2012; Munn et al., 2014), for assessing risk of bias in prevalence studies.

We will use a modified version of the *Navigation Guide* risk of bias tool (Lam et al., 2016c) that we developed specifically for Systematic Review 1 (Appendix D). We will assess risk of bias on the levels of the individual study and the entire body of evidence. As per our preliminary tool, we will assess risk of bias along five domains: (i) selection bias; (ii) performance bias; (iii) misclassification bias; (iv) conflict of interest; and (v) other biases. Risk of bias will be: “low”; “probably low”; “probably high”; “high” or “not applicable”. To judge the risk of bias in each domain, we will apply our a priori instructions (Appendix D).

All risk of bias assessors (PPMFK, SM and SN) will trial the tool until they have synchronized their understanding and application of each risk of bias domain, considerations and criteria for ratings. At least two study authors (out of: PPFMK, SM and SN) will then independently judge the risk of bias for each study by outcome and discuss any differences in order to come to consensus. A third author (MHWF) will resolve any conflicting judgments. We will present the findings of our risk of bias assessment for each eligible study in a standard ‘Risk of bias’ table (Higgins et al., 2011)*.* Our risk of bias assessment for the entire body of evidence will be presented in a standard ‘Risk of bias summary’ figure (Higgins et al., 2011).

#### 3.1.6 Synthesis of results

We will neither produce any summary measures, nor synthesise the evidence quantitatively. The included evidence will be presented in what could be described as an ‘evidence map’. All included data points from included studies will be presented, together with meta-data on the study design, number of participants, characteristics of population, setting, and exposure measurement of the data point.

#### 3.1.7 Quality of evidence assessment

There is no agreed method for assessing quality of evidence in systematic reviews of the prevalence of occupational and/or environmental risk factors We will adopt/adapt from the latest *Navigation Guide* instructions for grading (Lam et al., 2016c), including criteria (Appendix D). We will downgrade for the following five reasons from the Grading of Recommendations Assessment, Development and Evaluation (GRADE) approach: (i) risk of bias; (ii) inconsistency; (iii) indirectness; (iv) imprecision; and (v) publication bias (Schünemann et al., 2011). We will grade the evidence, using the three *Navigation Guide* quality of evidence ratings: “high”, “moderate” and “low” (Lam et al., 2016c). Within each of the relevant reasons for downgrading, we will rate any concern per reason as “none”, “serious” or “very serious”. We will start at “high” for non-randomized studies and will downgrade for no concern by nil, for a serious concern by one grade (−1), and for a very serious concern by two grades (−2). For example, if we have a serious concern for risk of bias in the body of evidence (−1) and a very serious concern for inconsistency (−2), We will downgrade the quality of evidence by a total of three grades (−3) from “high” to “low”. We will not up-grade or down-grade the quality of evidence for the three other reasons normally considered in GRADE assessments (i.e. large effect, dose-response and plausible residual confounding and bias), because we consider them irrelevant for prevalence estimates.

All quality of evidence assessors (HFVM, SM and SN) will trial the application of our instructions and criteria for quality of evidence assessment until their understanding and application is synchronized. At least two study authors (out of: HFVM, SM and SN) will independently judge the quality of evidence for the entire body of evidence by outcome. A third review author (CTJH or CHN) will resolve any conflicting judgments. In the systematic review, for each outcome, for each outcome, we will present our assessments of the risk for each GRADE domain, as well as an overall GRADE rating.

#### 3.1.8 Strength of evidence assessment

To our knowledge, no agreed method exists for rating strength of evidence in systematic reviews of prevalence studies, including those of occupational and/or environmental risk factors. We (HFVM, SN and SM) will rate the strength of the evidence for use as input data for estimating national-level exposure with the risk factor. Our rating will be based on a combination of the following four criteria: (i) quality of the entire body of evidence; (ii) population coverage of evidence (WHO regions and countries); (iii) confidence in the entire body of evidence; and (iv) other compelling attributes of the evidence that may influence certainty. We will rate the strength of the evidence as either “potentially sufficient” or “potentially inadequate” for use as input data (Appendix E).

### 3.2 Systematic Review 2

#### 3.2.1 Eligibility criteria

The PECO (Liberati et al., 2009) criteria are described below.

##### 3.2.1.1 Types of populations

We will include studies of working-age (≥15 year) workers in the formal and informal economy. Studies of children (aged <15 years) and unpaid domestic workers will be excluded. Participants residing in any WHO and/or ILO Member State and workers in any industrial setting and occupation will be included. Appendix F provides a briefer overview of the PECO criteria.

##### 3.2.1.2 Type of exposure

We will include studies that define exposure to occupational ergonomic risk factors in accordance with our standard definition (Table 1). We will include studies where exposure to occupational ergonomic risk factors was measured, whether objectively (e.g. by means of technology) or subjectively, including studies that used measurements by experts (e.g. scientists with subject matter expertise) and self-reports by the worker or workplace administrator or manager. If a study presents both objective and subjective measurements, then we will prioritize objective measurements. However, if we consider the subjective measure to be clearly preferable, then we will prioritize it and provide a justification for this prioritization in the systematic review. We will include studies with measures from any data source, including registry data. Studies from any year will be included.

##### 3.2.1.3 Type of comparators

The included comparator will be participants exposed to the theoretical minimum risk exposure level (Table 1). We will exclude all other comparators.

##### 3.2.1.4 Types of outcomes

We will include studies that defined osteoarthritis of hip or knee, and any selected other musculoskeletal diseases respectively, in accordance with our standard definition of these outcomes (Table 3). We will only include binary measures (present versus not present) of clinically assessed eligible osteoarthritis of hip or knee, and any selected other musculoskeletal diseases, respectively. Prevalence and incidence of eligible diseases will be included, but mortality will be excluded.

The following measurements of osteoarthritis, and any other musculoskeletal disease respectively, will be regarded as eligible:

i) Diagnosis by a physician.
ii) Hospital admission or discharge record.
iii) Other relevant administrative data (e.g. record of sickness absence or disability).
iv) Registry data of treatment for osteoarthritis of hip or knee and any selected other musculoskeletal disease, respectively.

All other measures will be excluded from this systematic review.

We will include objective measures of these eligible musculoskeletal diseases (e.g., measured by an occupational health and safety practitioner, such as an occupational physician or nurse, using a validated tool), as well as subjective measures (e.g., measured by a worker). If subjective and objective measures are presented, then we will prioritize objective measures.

##### 3.2.1.5 Types of studies

We will include studies that investigate the effect of exposure to any occupational risk factor on osteoarthritis of hip or knee, and any selected other musculoskeletal diseases respectively, for any years. Eligible study designs will be randomized controlled trials (including parallel-group, cluster, cross-over and factorial trials), cohort studies (both prospective and retrospective), case-control studies, and other non-randomized intervention studies (including quasi-randomized controlled trials, controlled before-after studies and interrupted time series studies). We included a broader set of observational study designs than is commonly included, because a recent augmented Cochrane Review of complex interventions identified valuable additional studies using such a broader set of study designs (Arditi et al., 2016). As we have an interest in quantifying risk and not in qualitative assessment of hazard (Barroga and Kojima, 2013), we will exclude all other study designs (e.g. uncontrolled before-and-after, cross-sectional, qualitative, modelling, case and non-original studies).

Records published in any year and any language will be included. Again, the search will be conducted using English language terms, so that records published in any language that present essential information (i.e. title and abstract) in English will be included. If a record is written in a language other than those spoken by the authors of this review or those of other reviews in the series, then the record will be translated into English. Published and unpublished studies will be included.

Studies conducted using unethical practices will be excluded from the review (e.g., studies that deliberately exposed humans to a known risk factor to human health).

##### 3.2.1.6 Types of effect measures

We will include measures of the relative effect of any exposure to occupational ergonomic risk factors on the prevalence or incidence of osteoarthritis of hip or knee and any selected other musculoskeletal disease respectively, compared with the theoretical minimum risk exposure level of no exposure. Effect estimates of mortality measures only will be excluded. We will include relative effect measures such as risk ratios and odds ratios for prevalence measures and hazard ratios for incidence measures (e.g., developed osteoarthritis and any musculoskeletal disease, respectively). Measures of absolute effects (e.g. mean differences in risks or odds) will be converted into relative effect measures, but if conversion is impossible, they will be excluded. To ensure comparability of effect estimates and facilitate meta-analysis, if a study presents an odds ratio, then we will convert it into a risk ratio, using the guidance provided in the Cochrane Collaboration's handbook for systematic reviews of interventions (Higgins and Green, 2011).

As shown in our logic framework (Fig. 1), we a priori consider the following variables to be potential effect modifiers of the effect of long working hours on stroke: country, age, sex, industrial sector, occupational group and formality of employment. We consider age, sex, socioeconomic position, body mass index, smoking status, comorbidity and sporting and/or leisure activities to be potential confounders. Potential mediators are tasks performed, load on the musculoskeletal system, psychosocial demands, social support, decision latitude, job control, job security, long working hours and work-related stress.

If a study presents estimates for the effect from two or more alternative models that have been adjusted for different variables, then we will systematically prioritize the estimate from the model that we consider best adjusted, applying the lists of confounders and mediators identified in our logic model (Fig. 1). We will prioritize estimates from models adjusted for more potential confounders over those from models adjusted for fewer. For example, if a study presents estimates from a crude, unadjusted model (Model A), a model adjusted for one potential confounder (Model B) and a model adjusted for two potential confounders (Model C), then we will prioritize the estimate from Model C. We will prioritize estimates from models unadjusted for mediators over those from models that adjusted for mediators, because adjustment for mediators can introduce bias. For example, if Model A has been adjusted for two confounders, and Model B has been adjusted for the same two confounders and a potential mediator, then we will choose the estimate from Model A over that from Model B. We prioritize estimates from models that can adjust for time-varying confounders that are at the same time also mediators, such as marginal structural models (Pega et al., 2016), over estimates from models that can only adjust for time-varying confounders, such as fixed-effects models (Gunasekara et al., 2014), over estimates from models that cannot adjust for time-varying confounding. If a study presents effect estimates from two or more potentially eligible models, then we will explain specifically why we prioritized the selected model.

#### 3.2.2 Information sources and search

##### 3.2.2.1 Electronic academic databases

At a minimum, we (JGD, HFVM, SM and SN) will search the six following electronic academic databases:

1. International Clinical Trials Register Platform (to 31 July 2018).
2. Ovid Medline with Daily Update (1 January 1946 to 31 July 2018).
3. PubMed (1 January 1946 to 31 July 2018).
4. EMBASE (1 January 1947 to 31 July 2018).
5. Web of Science with inclusion of three databases: Science Citation Index Expanded (1 January 1900 to 31 July 2018); Social Sciences Citation Index (1 January 1956 to 31 July 2018); Arts and Humanities Citation Index (1 January 1975 to 31 July 2018).
6. OSH UPDATE with inclusion of three databases: CISDOC (1 January 1974 to 31 July 2018); HSELINE (1 January 1977 to 31 July 2018); NIOSHTIC-2 (1 January 1977 to 31 July 2018).

The Ovid Medline search strategy for Systematic Review 2 is presented in Appendix G. To identify studies on musculoskeletal diseases, we have adopted or adapted several search terms or strings used in recent Cochrane Reviews on professional interventions for general practitioners on the management of musculoskeletal conditions (Tzortziou Brown et al., 2016) and ergonomic design and training for preventing work-related musculoskeletal diseases of the upper limb and neck in adults (Hoe et al., 2012). We will perform searches in electronic databases operated in the English language using a search strategy in the English language. We will adapt the search syntax to suit the other electronic academic and grey literature databases. When we are nearing completion of the review, we will search the PubMed database for the most recent publications (e.g., e-publications ahead of print) over the last six months. Any deviation from the proposed search strategy in the actual search strategy will be documented.

##### 3.2.2.2 Electronic grey literature databases

At a minimum, we (JGD, HFVM, SM and SN) will search the two following electronic grey literature databases:

1. OpenGrey (http://www.opengrey.eu/).
2. Grey Literature Report (http://greylit.org/).

##### 3.2.2.3 Internet search engines

We (JGD, HFVM, SM and SN) will also search the Google (www.google.com/) and Google Scholar (www.google.com/scholar/) Internet search engines and screen the first 100 hits for potentially relevant records.

##### 3.2.2.4 Organizational websites

The websites of the eight following international organizations and national government departments will be searched by PK, PPMFK and CC:

1. International Labour Organization (www.ilo.org/).
2. World Health Organization (www.who.int).
3. EU-OSHA (https://osha.europa.eu/en).
4. EUROSTAT (www.ec.europa.eu/eurostat/web/main/home).
5. China National Knowledge Infrastructure (CNKI) (http://www.cnki.net/).
6. Finnish Institute of Occupational Health (https://www.ttl.fi/en/).
7. National Institute of Occupational Safety and Health (NIOSH) of the United States of America, using the NIOSH data and statistics gateway (https://www.cdc.gov/niosh/data/).
8. International Ergonomics Association (http://www.iea.cc/).

##### 3.2.2.5 Hand-searching and expert consultation

We (CTJH, PPMFK, SM and SN) will hand-search for potentially eligible studies in:

• Reference lists of previous systematic reviews.
• Reference lists of all included study records.
• Study records published over the past 24 months in the three peer-reviewed academic journals with the largest number of included studies.
• Study records that have cited the included studies (identified in Web of Science citation database).
• Collections of the review authors.

Additional experts will be contacted with a list of included studies, with the request to identify potentially eligible additional studies.

#### 3.2.3 Study selection

Study selection will be carried out with Covidence (Babineau, 2014; *Covidence Systematic Review Software*) or the Rayyan Systematic Reviews Web App (Ouzzani et al., 2016). All study records identified in the search will be downloaded and duplicates will be identified and deleted. Afterwards, at least two review authors per health outcome area (out of: PK, PPFMK, FM, HFVM, SM and SN) will independently screen titles and abstracts (step 1) and then full texts (step 2) of potentially relevant records. A third review author (out of: CC, CTJH and CHN) will resolve any disagreements between the two review authors for the different health outcomes. If a study record identified in the literature search was authored by a review author assigned to study selection or if a assigned review author was involved the study that the study record reports, then the record will be re-assigned to another review author for study selection. The study selection will be documented in a flow chart in the systematic review, as per PRISMA guidelines (Liberati et al., 2009).

#### 3.2.4 Data extraction and data items

A data extraction form will be developed and trialed until data extractors reach convergence and agreement. At a minimum, two review authors (out of: PK, PPFMK, FM, HFVM, SM and SN) will extract data on study characteristics (including study authors, study year, study country, participants, exposure and outcome), study design (including summary of study design, comparator, epidemiological models used and effect estimate measure), risk of bias (including selection bias, reporting bias, confounding and reverse causation) and study context (e.g., data on contemporaneous exposure to other occupational risk factors potentially relevant for health loss from osteoarthritis and any other musculoskeletal disease, respectively). A third review author (out of: CC, MHWF and CHN) will resolve conflicts in data extraction, if any. Data will be entered into and managed with the RevMan 5.3 (*Review Manager (RevMan). Version 5.3,* 2014) or DistillerSR (EvidencePartner, 2017) computer softwares.

We will also extract data on potential conflict of interest in included studies. For each author and affiliated organization of each included study record, we will extract their financial disclosures and funding sources. We will use a modification of a previous method to identify and assess undisclosed financial interest of authors (Forsyth et al., 2014). Where no financial disclosure or conflict of interest statements are available, we will search the name of all authors in other study records gathered for this study and published in the prior 36 months and in other publicly available declarations of interests (Drazen et al., 2010a; Drazen et al., 2010b).

If relevant data are missing, we will contact the principal study author, using the email address provided in the principal study record, with a request to provide the missing data. If we do not receive a positive response from the study author, we will send follow-up emails twice, namely at two and four weeks.

#### 3.2.5 Risk of bias assessment

Standard risk of bias tools do not exist for systematic reviews for hazard identification in occupational and environmental health, nor for risk assessment. The five methods specifically developed for occupational and environmental health are for either or both hazard identification and risk assessment, and they differ substantially in the types of studies (randomized, observational and/or simulation studies) and data (e.g. human, animal and/or in vitro) they seek to assess (Rooney et al., 2016). However, all five methods, including the *Navigation Guide* (Lam et al., 2016c), assess risk of bias in human studies similarly (Rooney et al., 2016).

The *Navigation Guide* was specifically developed to translate the rigor and transparency of systematic review methods applied in the clinical sciences to the evidence stream and decision context of environmental health (Woodruff and Sutton, 2014), which includes workplace environment exposures and associated health outcomes. The guide is our overall organizing framework, and we will also apply its risk of bias assessment method in Systematic Review 2. The *Navigation Guide* risk of bias assessment method builds on the standard risk of bias assessment methods of the Cochrane Collaboration (Higgins et al., 2011) and the US Agency for Healthcare Research and Quality (Viswanathan et al., 2008). Some further refinements of the *Navigation Guide* method may be warranted (Goodman et al., 2017), but it has been successfully applied in several completed and ongoing systematic reviews (Johnson et al., 2014; Koustas et al., 2014; Lam et al., 2014; Vesterinen et al., 2014; Johnson et al., 2016; Lam et al., 2016b; Lam et al., 2017; Lam et al., 2016a). In our application of the *Navigation Guide* method, we will draw heavily on one of its latest versions, as presented in the protocol for an ongoing systematic review (Lam et al., 2016c). Should a more suitable method become available, we may switch to it.

We will assess risk of bias on the individual study level and on the body of evidence overall. The nine risk of bias domains included in the *Navigation Guide* method for human studies are: (i) source population representation; (ii) blinding; (iii) exposure assessment; (iv) outcome assessment; (v) confounding; (vi) incomplete outcome data; (vii) selective outcome reporting; (viii) conflict of interest; and (ix) other sources of bias. While two of the earlier case studies of the *Navigation Guide* did not utilize outcome assessment as a risk of bias domain for studies of human data (Johnson et al., 2014; Koustas et al., 2014; Lam et al., 2014; Vesterinen et al., 2014), all of the subsequent reviews have included this domain (Johnson et al., 2016; Lam et al., 2016b; Lam et al., 2017; Lam et al., 2016a; Lam et al., 2016c). Risk of bias or confounding ratings will be: “low”; “probably low”; “probably high”; “high” or “not applicable” (Lam et al., 2016c). To judge the risk of bias in each domain, we will apply a priori instructions (Appendix H), which we have adopted or adapted from an ongoing *Navigation Guide* systematic review (Lam et al., 2016c)). For example, a study will be assessed as carrying “low” risk of bias from source population representation, if we judge the source population to be described in sufficient detail (including eligibility criteria, recruitment, enrollment, participation and loss to follow up) and the distribution and characteristics of the study sample to indicate minimal or no risk of selection effects. The risk of bias at study level will be determined by the worst rating in any bias domain for any outcome. For example, if a study is rated as “probably high” risk of bias in one domain for one outcome and “low” risk of bias in all other domains for the outcome and in all domains for all other outcomes, the study will be rated as having a “probably high” risk of bias overall.

All risk of bias assessors (CC, CTJH, FM, HFVM, SM, PK, PPFMK, CHN and SN) will jointly trial the application of the risk of bias criteria until they have synchronized their understanding and application of the criteria. At least two study authors (out of: PK, PPFMK, FM, HFVM, SM and SN) will independently judge the risk of bias for each study by outcome. Where individual assessments differ, a third author (CC, CTJH or CHN) will resolve the conflict. In the systematic review, for each included study, we will report our study-level risk of bias assessment by domain in a standard ‘Risk of bias’ table (Higgins et al., 2011). For the entire body of evidence, we will present the study-level risk of bias assessments in a ‘Risk of bias summary’ figure (Higgins et al., 2011).

#### 3.2.6 Summary measures and synthesis of results

We will conduct meta-analyses separately for estimates of the effect on prevalence and incidence. If we find two or more studies with an eligible effect estimate (Table 2), two review authors (out of: PK, PPFMK, FM, HFVM, SM and SN) will independently investigate the clinical heterogeneity of the studies in terms of participants (including country, sex, age and industrial sector or occupation), level of risk factor exposure, comparator and outcomes. If we find that effect estimates differ considerably by country, sex and/or age, or a combination of these, then we will synthesise evidence for the relevant populations defined by country, sex and/or age, or combination thereof. Differences by country could include or be expanded to include differences by country group (e.g. WHO region or World Bank income group). If we find that effect estimates are clinically homogenous across countries, sexes and age groups, then we will combine studies from all of these populations into one pooled effect estimate that could be applied across all combinations of countries, sexes and age groups in the WHO/ILO joint methodology.

If we judge two or more studies for the relevant combination of country, sex and age group, or combination thereof, to be sufficiently clinically homogenous to potentially be combined quantitatively using quantitative meta-analysis, then we will test the statistical heterogeneity of the studies using the I^2^ statistic (Figueroa, 2014). If two or more clinically homogenous studies are found to be sufficiently homogenous statistically to be combined in a meta-analysis, we will pool the risk ratios of the studies in a quantitative meta-analysis, using the inverse variance method with a random effects model to account for cross-study heterogeneity (Figueroa, 2014). The meta-analysis will be conducted in RevMan 5.3, but the data for entry into these programmes may be prepared using another recognized statistical analysis programme, such as Stata. We will not quantitatively combine data from studies with different designs (e.g. combining cohort studies with case-controls studies), nor unadjusted and adjusted models. We will only combine studies that we judge to have a minimum acceptable level of adjustment for confounders. If quantitative synthesis is not feasible, then we will synthesise the study findings narratively and identify the estimates that we judged to be the highest quality evidence available.

#### 3.2.7 Additional analyses

If we source micro-data on exposure, outcome and potential confounding variables, we may conduct meta-regressions to adjust optimally for potential confounders.

If there is evidence for differences in effect estimates by country, sex, age, industrial sector and/or occupation, or by a combination of these variables, then we will conduct subgroup analyses by the relevant variable or combination of variables, as feasible. Where both studies on workers in the informal economy and studies on workers in the formal economy are included, we will conduct subgroup analyses by formality of economy. Findings of these subgroup analyses, if any, will be used as parameters for estimating burden of disease specifically for relevant populations defined by these variables. We will also conduct subgroup analyses by study design (e.g. randomized controlled trials versus cohort studies versus case-control studies).

We will perform a sensitivity analyses that will include only studies judged to be of “low” or “probably low” risk of bias from conflict of interest; judged to be of “low” or “probably low” risk of bias; and with documented or approximated ICD-10 diagnostic codes. We may also conduct a sensitivity analysis using an alternative meta-analytic model, namely the inverse variance heterogeneity (IVhet) model (Doi et al., 2017).

#### 3.2.8 Quality of evidence assessment

We will assess quality of evidence using a modified version of the *Navigation Guide* quality of evidence assessment tool (Lam et al., 2016c). The tool is based on the GRADE approach (Schünemann et al., 2011) adapted specifically to systematic reviews in occupational and environmental health (Morgan et al., 2016). Should a more suitable method become available, we may switch to it.

We (out of: PK, PPFMK, FM, HFVM, SM and SN) will assess quality of evidence for the entire body of evidence by outcome. We will adopt or adapt the latest *Navigation Guide* instructions (Appendix D) for grading the quality of evidence (Lam et al., 2016c). We will downgrade the quality of evidence for the following five GRADE reasons: (i) risk of bias; (ii) inconsistency; (iii) indirectness; (iv) imprecision; and (v) publication bias. If our systematic review includes ten or more studies, we will generate a funnel plot to judge concerns on publication bias. If it includes nine or fewer studies, we will judge the risk of publication bias qualitatively. To assess risk of bias from selective reporting, protocols of included studies, if any, will be screened to identify instances of selective reporting.

We will grade the evidence, using the three *Navigation Guide* standard quality of evidence ratings: “high”, “moderate” and “low” (Lam et al., 2016c). Within each of the relevant domains, we will rate the concern for the quality of evidence, using the ratings “none”, “serious” and “very serious”. As per *Navigation Guide*, we will start at “high” for randomized studies and “moderate” for observational studies. Quality will be downgrade for no concern by nil grades (0), for a serious concern by one grade (−1) and for a very serious concern by two grades (−2). We will up-grade the quality of evidence for the following other reasons: large effect, dose-response and plausible residual confounding and bias. For example, if we have a serious concern for risk of bias in a body of evidence consisting of observational studies (−1), but no other concerns, and there are no reasons for upgrading, then we will downgrade its quality of evidence by one grade from “moderate” to “low”.

#### 3.2.9 Strength of evidence assessment

We (out of: PK, PPFMK, FM, HFVM, SM and SN) will apply the standard *Navigation Guide* methodology (Lam et al., 2016c) to rate the strength of the evidence. The rating will be based on a combination of the following four criteria: (i) quality of the body of evidence; (ii) direction of the effect; (iii) confidence in the effect; and (iv) other compelling attributes of the data that may influence our certainty. The ratings for strength of evidence for the effect of exposure to occupational ergonomic risk factors on osteoarthritis, and any other musculoskeletal disease respectively, will be “sufficient evidence of harmfulness”, “limited evidence of harmfulness”, “inadequate evidence of harmfulness” and “evidence of lack of harmfulness” (Appendix I).

## Financial support

All authors are salaried staff members of their respective institutions. The publication was prepared with financial support from the 10.13039/100004423World Health Organization cooperative agreement with the Centres for Disease Control and Prevention National Institute for Occupational Safety and Health of the United States of America on implementing Resolution WHA 60.26 “Workers' Health: Global Plan of Action” (Grant 1 E11 OH0010676-02).

## Sponsors

The sponsors of this systematic review are the 10.13039/100004423World Health Organization and the 10.13039/100008633International Labour Organization.

## Author contributions

II, NL, FP and APÜ had the idea for the systematic review. II, NL, FP and YU gathered the review team. FP led and all authors contributed to the development of the standard methodology for all systematic reviews in the series. FP led and all authors contributed to the development and writing of the standard template for all protocols in the series. CTJH and MHWF are the lead reviewers of this systematic review. CTJH, MHWF, and HFVM wrote the first draft of this protocol, using the protocol template prepared by FP, and CC, JGD, PDL, MHWF, CTJH, PK, PPFMK, SM, FM, SN, CHN, JO, FP, KP, APŰ, YU and HFVM made substantial contributions to the revisions of the manuscript. The search strategy was developed and piloted by JGD. HFVM, CHN, and FP are experts in epidemiology, PPFMK and HFVM are occupational ergonomists, and HFVM, CTJH, and FP are experts in systematic review methodology. FP coordinated all inputs from the World Health Organization, International Labour Organization and external experts and ensured consistency across the systematic reviews of the series. CTJH and MHWF are the guarantors of the systematic reviews.

## Appendices A, B, C, D, E, F, G, H and I Supplementary data

Supplementary material Image 1
