# Supplementary material for: WHO/ILO work-related burden of disease and injury: Protocol for systematic reviews of exposure to occupational ergonomic risk factors and of the effect of exposure to occupational ergonomic risk factors on osteoarthritis of hip or knee and selected other musculoskeletal diseases
Source: Environ Int. 2019 Apr;125:554–66. doi: 10.1016/j.envint.2018.09.053 (PMC7794864; doi:10.1016/j.envint.2018.09.053)
Supplement: Supplementary file 1 — Supplementary material [file mmc1.docx]

**Appendices**

[Appendix A: Overview of inclusion and exclusion criteria, Systematic Review 1 2](#_Toc520220914)

[Appendix B: Proposed Ovid Medline search strategy, Systematic Review 1 3](#_Toc520220915)

[Appendix C: Rate risk of bias, Systematic Review 1 6](#_Toc520220916)

[Appendix D: Instructions for grading the quality of evidence, Systematic Reviews 1 and 2 15](#_Toc520220917)

[Appendix E: Rate the strength of evidence, Systematic Review 1 23](#_Toc520220918)

[Appendix F: Overview of inclusion and exclusion criteria, Systematic Review 2 24](#_Toc520220919)

[Appendix G: Proposed Ovid Medline search strategy, Systematic Review 2 25](#_Toc520220920)

[Appendix H: Rate risk of bias, Systematic Review 2 30](#_Toc520220921)

[Appendix I: Rate the strength of evidence, Systematic Review 2 41](#_Toc520220922)

[Reference list for all appendices 42](#_Toc520220923)

# Appendix A: Overview of inclusion and exclusion criteria, Systematic Review 1

***Include***

1. Quantitative studies of any design, including cross-sectional studies, on the prevalence of exposure to occupational ergonomic risk factors for the years 2005 to 2018

2. Studies of working-age (≥15 year) workers in the formal or informal economy

3. Studies of workers residing in any Member States of WHO and/or ILO and working any industrial setting or occupation

4. Studies on the prevalence of exposure to occupational ergonomic risk factors, if the prevalence is disaggregated by country, sex, age and industrial sector or occupation. Criteria may be revised in order to identify optimal data disaggregation to enable subsequent estimation of the burden of disease

5. Studies published between 1 January 2005 and 31 July 2018 in any language

***Exclude***

1. Studies of unpaid domestic workers

2. Studies of children (aged ˂15 years)

3. Studies with data only on years before 2005

4. Qualitative, modelling, and case studies, as well as non-original studies without quantitative data (e.g. letters, commentaries and perspectives)

# Appendix B: Proposed Ovid Medline search strategy, Systematic Review 1

| **ELBOW** |  |  |
| --- | --- | --- |
|  | 1 | (worka* or worke* or workg* or worki* or workl* or workp* or work capacity or work disabilit* or work abilit* or at work or work exposure or work related or workers or job* or employee or staff or personnel or occupation or occupations or occupational or outdoor work* or day shift* or night shift* or shift work* or vocational rehabilitation or sick leave or absenteeism or sickness absen* or absente* or presente* or "return to work" or vocational reintegration or retirement or pension or employment or unemployed or unemployment or work status or industries or industrial sector or volunteer* or voluntary worker* or repetitive work).ab,kf,ti. |
|  | 2 | posture/ or evaluation studies as topic/ or exp causality/ or risk assessment/ or time factors/ or lifting/ or vibration/ or weight bearing/ or cumulative trauma disorders/ |
|  | 3 | ((posture adj3 wrist?) or (elevat* adj3 arm?) or "computer use" or "computer usage" or kneel* or climbing or repetition strain or repetitive strain or repetitive motion or repetitive movement? or (repetitive adj5 activit*) or repetition motion or overuse or biomechanical or lift* or manual work* or manual labo* or carry* or elevate? or arm force or hand force or hand vibration or arm vibration or (Handling adj2 (heavy or load? or weight)) or Handgrip force or Vibrating tool? or Handling tools or risk? or course? or etiolog* or causality or exposure?).ab,kf,ti. |
|  | 4 | 2 or 3 |
|  | 5 | elbow/ or hand arm vibration syndrome/ or exp cumulative trauma disorders/ or exp arm injuries/ |
|  | 6 | (elbow? or medial epicondylitis or Lateral Epicondylitis or Olecranon bursitis).ab,kf,ti. |
|  | 7 | 5 or 6 [anatomy \| relevant disorders] |
|  | 8 | and/1,4,7 |
|  | 9 | limit 8 to yr="2005-2018" |
|  | 10 | dedup 9 |
|  |  |  |
| **KNEE** |  |  |
|  | 1 | (worka* or worke* or workg* or worki* or workl* or workp* or work capacity or work disabilit* or work abilit* or at work or (work adj4 expos*) or work related or job* or employ* or or unemploy* or staff or personnel or occupation* or outdoor work* or day shift* or night shift* or shift work* or vocational rehabilitation or sick leave or sickness absen* or absente* or presente* or "return to work" or vocational reintegration or retirement* or pension* or work status or industries or industrial sector or volunteer* or voluntary worker* or repetitive work*).ab,kf,ti. |
|  | 2 | evaluation studies as topic/ or exp causality/ or risk assessment/ or time factors/ or lifting/ or weight bearing/ or cumulative trauma disorders/ |
|  | 3 | (kneel* or squatting or climbing or physical work or repetition strain or repetitive strain or repetitive motion or repetition motion or (repetitive adj5 activit*) or overuse or biomechanical or lift* or manual work* or manual labo* or carry* or risk? or course? or etiolog* or causality or exposure?).ab,kf,ti. |
|  | 4 | 2 or 3 [evaluation studies \| (known) risk] |
|  | 5 | knee/ or knee joint/ or "menisci, tibial"/ or exp cumulative trauma disorders/ or chondromalacia patellae/ or exp knee injuries/ or tibial meniscus injuries/ |
|  | 6 | (knee? or Superior tibiofibular joint or proximal tibiofibular articulation or Prepatellar bursitis or chondromalacia patella* or lateral menisc* or tibial menisc* or medial meniscus or meniscus injur* or meniscus disorder?).ab,kf,ti. |
|  | 7 | or/5-6 [anatomy \| relevant diseases] |
|  | 8 | and/1,4,7 |
|  | 9 | limit 8 to yr="2005-2018" |
|  | 10 | ..dedup 9 |
|  |  |  |
| **SHOULDER** |  |  |
|  | 1 | (worka* or worke* or workg* or worki* or workl* or workp* or work capacity or work disabilit* or work abilit* or at work or work exposure or work related or workers or job* or employee or staff or personnel or occupation or occupations or occupational or outdoor work* or day shift* or night shift* or shift work* or vocational rehabilitation or sick leave or absenteeism or sickness absen* or absente* or presente* or "return to work" or vocational reintegration or retirement or pension or employment or unemployed or unemployment or work status or industries or industrial sector or volunteer* or voluntary worker* or repetitive work).ab,kf,ti. |
|  | 2 | posture/ or evaluation studies as topic/ or exp causality/ or risk assessment/ or time factors/ or lifting/ or vibration/ or weight bearing/ or exp age factors/ |
|  | 3 | (lift* or carry* or elevate? or work above or risk? or course? or etiolog* or causality or exposure?).ab,kf,ti |
|  | 4 | 2 or 3 [evaluation studies \| (known) risk] |
|  | 5 | shoulder joint/ or rotator cuff/ or shoulder pain/ or shoulder impingement syndrome/ or hand arm vibration syndrome/ or exp cumulative trauma disorders/ |
|  | 6 | (((arm? or shoulder? or upper limb? or upper extremit*) adj3 (vibration or repetitive)) or shoulder pain or (shoulder? adj5 (complaint? or disorder? or exertion or flexion or repetitive)) or shoulder impingement or rotator cuff syndrome or cumulative trauma disorder?).ab,kf,ti |
|  | 7 | or/5-6 [anatomy \| relevant diseases] |
|  | 8 | and/1,4,7 |
|  | 9 | limit 8 to yr="2005-2018" |
|  | 10 | ..dedup 9 |
|  |  |  |
| **HIP** |  |  |
|  | 1 | (worka* or worke* or workg* or worki* or workl* or workp* or work capacity or work disabilit* or work abilit* or at work or work exposure or work related or workers or job* or employee or staff or personnel or occupation or occupations or occupational or outdoor work* or day shift* or night shift* or shift work* or vocational rehabilitation or sick leave or absenteeism or sickness absen* or absente* or presente* or "return to work" or vocational reintegration or retirement or pension or employment or unemployed or unemployment or work status or industries or industrial sector or volunteer* or voluntary worker* or repetitive work).ab,kf,ti. |
|  | 2 | evaluation studies as topic/ or exp causality/ or risk assessment/ or time factors/ or lifting/ or weight bearing/ or workload/ |
|  | 3 | (lift* or carry* or workload? or work load? or risk? or course? or etiolog* or causality or exposure?).ab,kf,ti |
|  | 4 | 2 or 3 [evaluation studies \| (known) risk] |
|  | 5 | Osteoarthritis, Hip/ or (hip/ and osteoarthritis/) |
|  | 6 | coxarthr* or ((osteoarthr* or arthr* or oa) adj2 (cox? or hip?)) or (joint disease adj3 (cox? or hip?)).ab,kf,ti |
|  | 7 | or/5-6 [anatomy \| relevant diseases] |
|  | 8 | and/1,4,7 |
|  | 9 | limit 8 to yr="2005-2018" |
|  | 10 | ..dedup 9 |

# Appendix C: Rate risk of bias, Systematic Review 1

**Tool development**

Departing from the *Navigation Guide* (Woodruff and Sutton 2014) risk of bias tool (Lamet al. 2016), we first selected the five relevant risk of bias domains (i.e., selection bias, performance bias, exposure measurement, conflict of interest, and other bias), and we adopted or adapted the relevant considerations and criteria for ratings. Second, we integrated key considerations from the GATHER Guidelines (The GATHER Working Group 2016) and existing checklists for prevalence studies (Hoyet al. 2012; Munnet al. 2014). Third, we integrated relevant criteria for ratings for the exposure measurement domain from the US Office of Health Assessment and Translation / National Toxicology Program risk of bias tool (Rooney 2015). Finally, systematic review methodologists from all systematic reviews in the series (Descathaet al. 2018; Godderiset al. 2018; Liet al. Accepted; Mandrioliet al. 2018; Pauloet al. Under review; Rugulieset al. Accepted; Teixeiraet al. Accepted; Tenkateet al. Accepted) jointly improved the preliminary tool, and they will further refine and test it over the course of the systematic reviews. If a more suitable method becomes available over the course of the systematic review, then we may switch to it.

***Instructions***

- *Please evaluate each individual study for the following five risk of bias domains, indicated by one key question each.*
- *For each risk of bias domain, please rate the risk as “low”; “probably low”; “probably high”; “high”; or “not applicable”.*
- *For each risk of bias domain, please provide a justification for your rating.*
- *If there is empirical evidence or other knowledge that informs the direction of bias, please include this in your answer. However, if there is not enough information to robustly indicate direction of a potential bias, please do not guess the direction of the bias.*
- *Some internal validity issues could potentially be appropriately captured in considerations for several different risk of bias domains. In this situation, please select the single most appropriate domain to evaluate this potential bias, to avoid double-counting the same internal validity concern.*

***1. Is there a risk that exposures captured in the study sample do not represent exposures in the target population in a manner that might introduce selection bias?***

The target population is defined as the population, for which study investigators aim to assess exposures.

Examples of considerations for this risk of bias domain include:

1. the study sample is an adequate representation of the target population
2. the study sample and the target population have similar characteristics (e.g., sociodemographic characteristics, occupation and disease status)
3. participant inclusion and exclusion were appropriately defined
4. the sampling frame is representative of the target population
5. either the study sample was drawn at random, or a census was undertaken
6. the proportion of persons invited to participate in the study who did participate in the study was acceptable
7. the proportion of persons invited to participate in the study who did participate in the study was comparable across exposure levels
8. the reasons for non-participation in the study were acceptable
9. the proportion of study participant who participated in the exposure assessment was acceptable
10. the proportion of study participant who participated in the exposure assessment was comparable across exposure levels
11. the reasons for non-participation in exposure assessment were acceptable

If feasible, please also access and consider information reported in other study records from the study.

Criteria for a rating of LOW risk of bias (i.e., the answer to Question 1 above is: “No”):

- 1. The descriptions of the target population, inclusion/exclusion criteria, recruitment and enrolment procedures (including sampling frame), participation/response rates are sufficiently detailed, and adequate data were supplied on the distribution of relevant study sample and population characteristics, so that you judge the risk of selection effects to be low.

OR

- 1. The descriptions and/or data as indicated in “a)” above do suggest the potential for selection effects. However, adequate information was given that you can judge any potential selection effects as *not* differential across sub-groups defined by exposure levels.

OR

- 1. The descriptions and/or data as indicated in “a)” above do suggest the potential for selection effects, and there was no information suggesting that potential selection effects were *not* differential across sub-groups defined by exposure levels. However, drivers of selection effects were well-understood, these drivers of selection effects were measured in the data set, and appropriate post-hoc statistical methods were used to control for potential selection bias.

Criteria for the rating of PROBABLY LOW risk of bias (i.e., the answer to Question 1 above is: “Probably No”):

There is insufficient information about participant selection to permit you to judge the risk of bias to be low. However, indirect evidence suggests that inclusion/exclusion criteria, recruitment and enrolment procedures, and participation/response rates were consistent across groups as described by the criteria for a rating of low risk of bias.

Criteria for the rating of PROBABLY HIGH risk of bias (i.e., the answer to Question 1 above is: “Probably Yes”):

There is insufficient information about participant selection to permit you to judge the risk of bias to be high. However, indirect evidence suggests that inclusion/exclusion criteria, recruitment and enrolment procedures, and participation/response rates were inconsistent across groups, as described by the criteria for a rating of high risk of bias.

Criteria for the rating of HIGH risk of bias (i.e., i.e., the answer to Question 1 above is: “Yes”):

1. The descriptions of the target population, inclusion/exclusion criteria, recruitment and enrolment procedures, participation/response rates and/or data on the distribution of relevant study sample and population characteristics suggest that the risk of selection effects was substantial;

AND

1. No information was provided to indicate that potential selection effects were *not* differential across sub-groups defined by exposure level;

AND

1. No appropriate post-hoc techniques were used to control for potential selection bias.

Criteria for the rating of NOT APPLICABLE (Question 1 is not applicable to the study):

There is evidence that participant selection is not an element of study design capable of introducing risk of bias in the study.

***2. Were exposure assessors and other study personnel blinded to relevant participant characteristics (e.g. occupation and/or disease status) or could exposure assessors’ or other study personnel’s prior knowledge of participant characteristics have influenced the exposure assessment in a manner that might introduce performance bias?***

Criteria for a rating of LOW risk of bias (i.e., the answer to Question 2 above is: “No”):

1. Exposure assessors and study personnel were blinded to relevant participant characteristics (e.g. occupation and/or disease status), and the blinding was probably not broken.

OR

1. Exposure assessors and study personnel were not blinded to relevant participant characteristics. However, this lack of blinding is unlikely to have influenced the exposure measurement (e.g., it was likely that exposure was systematically assessed similarly across sub-groups defined by participant characteristic);

Criteria for the rating of PROBABLY LOW risk of bias (i.e., the answer to Question 2 above is: “Probably No”):

The information on blinding is insufficient to permit a rating of low risk of bias. However, indirect evidence suggests that the exposure assessors and study personnel were adequately blinded, as described by the criteria for a rating of low risk of bias.

Criteria for the rating of PROBABLY HIGH risk of bias (i.e., the answer to Question 2 above is: “Probably Yes”):

The information on blinding is insufficient to permit a rating of high risk of bias. However, indirect evidence suggests that the exposure assessors and study personnel were not adequately blinded, as described by the criteria for a rating of high risk of bias.

Criteria for the rating of HIGH risk of bias (i.e., the answer to Question 2 above is: “Yes”):

- Exposure assessors and study personnel were not at all blinded or incompletely blinded, and the exposure measures are likely to be influenced by the lack of blinding (e.g., exposure was systematically assessed differentially for sub-groups defined by participant characteristics).

OR

- Blinding of exposure assessors and study personnel was attempted, but may have been broken, and the breaking of the blinding may have introduced bias.

Criteria for the rating of NOT APPLICABLE (Question 2 is not applicable to the study):

There is evidence that blinding is not an element of study design capable of introducing risk of bias in the study.

***3. Is there a risk that the methods for assessing exposure might consistently over- or under-estimate exposure in a manner that might introduce misclassification bias?***

*The following list of potential considerations is a collection of factors that may potentially systematically influence the internal validity of the exposure assessment (not those that may randomly affect overall study results). These considerations should be interpreted only as suggested ones and not as a scoring or a checklist.*

***List of potential considerations****:*

Quality of exposure measurement:

1. Was the case definition used in the exposure measurement appropriate and consistently applied?
2. Was the exposure self-reported by the participant for whom the exposure was measured?
3. In self-reported exposure measurement, were study participants aware of their own health status?
4. Was the exposure measure in other ways dependent on study participants’ characteristics (e.g. occupation and/or disease status)?
5. Was the exposure assessment based on individual exposure measurements (e.g. personal dosimetry) or aggregate environmental/ambient/area exposure assessment?
6. Was the exposure assessment influenced by non-occupational exposure (e.g., the environmental concentration of an occupational risk factor)?
7. Did the exposure assessment appropriately take into account the adoption of protective gear and other preventive strategies?
8. If technical devices were used for exposure assessment, did the technical requirements of the devices used to perform the exposure measurement meet specific and established standards (e.g. devices were properly calibrated)?
9. Has the exposure measurement been validated for relevant populations and settings?

Data collection:

1. Were data collected directly from the participants (as opposed to by proxy)?
2. Was the same data collection mode used for measuring exposure among all study participants?

Sample size

1. Was the size of the total study sample appropriate?
2. Was the size of the study sub-samples for each exposure level was appropriate

Validity of prevalence calculation:

1. Was the length of the shortest prevalence period for the assessed exposure appropriate (e.g. the shortest prevalence period covered was at least one full working day)?
2. Were the numerator and denominator for the prevalence estimate appropriate?

Missing data:

1. Were missing data appropriately accounted for (e.g., missing data were appropriately imputed)?

Criteria for a rating of LOW risk of bias (i.e., the answer to Question 3 above is: “No”):

The reviewers judge that there is low risk of exposure misclassification, i.e.:

1. There is high confidence in the accuracy of the exposure measurement method (e.g., the methods have been tested for validity and reliability in measuring the targeted exposure). In other words, *direct* evidence suggests that exposure was consistently assessed (i.e., under the same method and time-frame) using well-established methods that directly measure exposure; the exposure measurement assessed relevant levels of the exposure over relevant reporting periods.

OR

1. Less-established or less direct exposure measurements are validated against well-established or direct methods. For example, the exposure was assessed using less-established methods that directly measure exposure, but the methods are validated against well-established methods; exposure was assessed for a relevant reporting period; the exposure measurement assessed relevant levels of the exposure over relevant reporting periods.

Criteria for the rating of PROBABLY LOW risk of bias (i.e., the answer to Question 3 above is: “Probably No”):

There is insufficient information about the exposure measurement methods to permit a rating of low risk of bias. However, there is *indirect* evidence that exposure measurement methods were accurate, as described by the criteria for a rating of low risk of bias:

1. *Indirect* evidence suggests that the exposure was consistently assessed using well-established methods that directly measure exposure

OR

1. Exposure was assessed using indirect measures (e.g., questionnaire or occupational exposure measurement by a certified occupational health and safety professional) that have been validated or empirically shown to be consistent with methods that directly measure exposure (i.e., inter-methods validation: one method vs. another). And the exposure measurement assessed relevant levels of the exposure over relevant reporting periods.

Criteria for the rating of PROBABLY HIGH risk of bias (i.e., the answer to Question 3 above is: “Probably Yes”):

There is insufficient information about the exposure measurement methods to permit a rating of high risk of bias, but there is indirect evidence that suggests that methods were not robust, as described by the criteria for a rating of high risk of bias:

1. *Indirect* evidence that the exposure was assessed using poorly validated methods that directly measure exposure.

OR

1. There is evidence that the exposure was assessed using indirect measures that have not been validated or empirically shown to be consistent with methods that directly measure exposure (e.g., questionnaire, job-exposure matrix or self-report without validation).

OR

1. Insufficient information is provided about the exposure measurement method, including validity and reliability, but there is no evidence for concern about the exposure measurement method used.

Criteria for the rating of HIGH risk of bias (i.e., the answer to Question 3 above is: “Yes”):

- - - *Direct* evidence suggests that the exposure was assessed using methods with poor validity.

OR

- - - Evidence suggests exposure misclassification (e.g., differential recall of self-reported exposure).

OR

- - - It is unclear how exposure measurement was obtained.

Criteria for the rating of NOT APPLICABLE (Question 3 is not applicable to the study):

There is evidence that exposure measurement methods are not capable of introducing risk of bias in the study.

***4. Did the study receive any support from a company, study author or other entity with a potential financial interest in the exposures assessed?***

Criteria for a judgment of LOW risk of bias Financial conflicts of interest are defined per the July 2010 version of the International Committee of Medical Journal Editors uniform disclosure form for potential conflicts of interest and included: concurrent or former board membership, concurrent or former consultancy work, concurrent or former industry employment, expert testimony, industry grants (issued or pending), payment for lectures including service on speakers bureaus, payment for manuscript preparation, patents (planned, pending, or issued), royalties, payment for development of educational presentations, stock or stock options, and travel reimbursement, or other relations with relevant industries (ICMJE 2010).

Criteria for a rating of LOW risk of bias (i.e., the answer to Question 4 above is: “No”):

The study did not receive support from a company, study author or other entity that had a financial interest in the outcome of the study. Examples include the following:

- Funding source is limited to government, non-profit organizations or academic grants funded by government, foundations and/or non-profit organizations;
- Treatment used in the study (if any) was purchased from a supplier;
- Company-affiliated staff are not mentioned in the acknowledgements section;
- Authors were not employees of a company with a financial interest in the outcome of the study;
- Company with a financial interest in the outcome of the study was not involved in the design, conduct, analysis or reporting of the study and authors had complete access to the data;
- Study authors make a claim denying conflicts of interest;
- Study authors are unaffiliated with companies with financial interest, and there is no reason to believe a conflict of interest exists;
- All study authors are affiliated with a government agency (are prohibited from involvement in projects, for which there is a potential conflict of interest).

Criteria for the rating of PROBABLY LOW risk of bias (i.e., the answer to Question 4 above is: “Probably No”):

There is insufficient information to permit a rating of low risk of bias, but there is indirect evidence which suggests the study was free of support from a company, study author, or other entity having a financial interest in the outcome of the study, as described by the criteria for a rating of low risk of bias.

Criteria for the rating of PROBABLY HIGH risk of bias (i.e., the answer to Question 4 above is: “Probably Yes”):

There is insufficient information to permit a rating of high risk of bias, but there is indirect evidence which suggests the study was not free of support from a company, study author, or other entity having a financial interest in the outcome of the study, as described by the criteria for a rating of high risk of bias.

Criteria for the rating of HIGH risk of bias (i.e., the answer to Question 4 above is: “Yes”):

The study received support from a company, study author or other entity that had a financial interest in the outcome of the study. Examples of support include:

- Research funds;
- Chemicals, equipment or testing provided at no cost;
- Writing services;
- Author/staff from the study was an employee or otherwise affiliated with a company with a financial interest;
- Company limited author access to the data;
- Company was involved in the design, conduct, analysis or reporting of the study;
- Study authors claim a conflict of interest

Criteria for the rating of NOT APPLICABLE (Question 4 is not applicable to the study):

There is evidence that conflicts of interest are not capable of introducing risk of bias in the study.

***5. Did the study appear to have other problems that could put it at a risk of bias?***

Criteria for a rating of LOW risk of bias (i.e. the answer to Question 5 above is: “No”):

The study appears to be free of other sources of bias.

Criteria for the rating of PROBABLY LOW risk of bias (i.e., the answer to Question 5 above is: “Probably No”):

There is insufficient information to permit a rating of low risk of bias, but there is indirect evidence which suggests the study was free of other threats to validity.

Criteria for the rating of PROBABLY HIGH risk of bias (i.e., the answer to Question 5 above is: “Probably Yes”):

There is insufficient information to permit a rating of high risk of bias, but there is indirect evidence which suggests the study was not free of other threats to validity, as described by the criteria for a rating of high risk of bias.

Criteria for the rating of HIGH risk of bias (i.e., the answer to Question 5 above is: “Yes”):

There is at least one important other risk of bias. For example, the study:

- Had a potential source of bias related to the specific study design used;
- Stopped early due to some data-dependent process (including a formal-stopping rule);
- The conduct of the study is affected by interim results (e.g. recruiting additional participants from a sub-group showing greater or lesser effect);
- Has been claimed to have been fraudulent; and/or
- Had some other risk of bias.

# Appendix D: Instructions for grading the quality of evidence, Systematic Reviews 1 and 2

Most of the text from these instructions and criteria for judging risk of bias has been adopted verbatim or adapted from one of the latest *Navigation Guide* (Woodruff and Sutton 2014) systematic reviews (Lamet al. 2016).

1. ***Grading Quality***

Each of the categories to consider in downgrading or upgrading the evidence is described in detail below. Please record your results on the chart at the end of each category, including a brief explanation for your ratings.

**Category 1. Quality of Study Limitations (Risk of Bias)**

Possible ratings: 0=no change; -1 or -2 downgrade 1 or 2 levels

The evidence from studies can be rated down if most of the relevant evidence comes from studies that suffer from a high risk of bias. Risk of bias is rated by outcome across studies. Study limitations for each outcome for individual studies and across studies are summarized in the heat maps. GRADE outlines the following principles for moving from risk of bias in individual studies to rating quality of evidence across studies.

1. In deciding on the overall quality of evidence, one does not average across studies (for instance if some studies have no serious limitations, some serious limitations, and some very serious limitations, one does not automatically rate quality down by one level because of an average rating of serious limitations). Rather, judicious consideration of the contribution of each study, with a general guide to focus on the high-quality studies is warranted.^^[[1]](#footnote-2)^^
2. This judicious consideration requires evaluating the extent to which each study contributes toward the estimate of magnitude of effect. The contribution that each study makes will usually reflect study sample size and number of outcome events. Larger studies with many events will contribute more, much larger studies with many more events will contribute much more.
3. One should be conservative in the judgment of rating down. That is, one should be confident that there is substantial risk of bias across most of the body of available evidence before one rates down for risk of bias.
4. The risk of bias should be considered in the context of other limitations. If, for instance, reviewers find themselves in a close-call situation with respect to two quality issues (risk of bias and, say, precision), GRADE suggests rating down for at least one of the two.
5. Notwithstanding the first four principles, reviewers will face close-call situations. You should acknowledge that you are in such a situation, make it explicit why you think this is the case, and make the reasons for your ultimate judgment apparent.

| **Rating for Risk of Bias (Study Limitations)**  0 no change  -1 decrease quality 1 level  -2 decrease quality 2 levels | | **Rationale for your judgment** |
| --- | --- | --- |
| Human |  |  |

**Category 2. Indirectness of Evidence**

Possible ratings: 0=no change; -1 or -2 downgrade 1 or 2 levels

Quality of evidence (your confidence in estimates of effect) may decrease when substantial differences exist between the population, exposure, or outcomes measured in the research studies under consideration in the review.

Evidence is direct when it directly compares the exposures in which we are interested in the populations in which we are interested and measures outcomes important to the study question (in GRADE the outcomes must be important to patients).

Based on GRADE, evidence can be indirect in one of three ways.^^[[2]](#footnote-3)^^

- 1. The population studied differs from the population of interest (the term applicability is often used for this form of indirectness). GRADE states that in general, one should not rate down for population differences unless one has compelling reason to think that the biology in the population of interest is so different than the population tested that the magnitude of effect will differ substantially. According to GRADE, most often, this will not be the case.
  2. The intervention (exposure) tested may differ from the exposure of interest, i.e., a difference in the chemical, route and/or dose. Decisions regarding indirectness of populations and exposure depend on an understanding of whether biological or social factors are sufficiently different that one might expect substantial differences in the magnitude of effect. GRADE also states, “As with all other aspects of rating quality of evidence, there is a continuum of similarity of the intervention that will require judgment. It is rare, and usually unnecessary, for the intended populations and interventions to be identical to those in the studies, and we should only rate down if the differences are considered sufficient to make a difference in outcome likely.”
  3. Outcomes may differ from those of primary interest; for instance, surrogate outcomes that are not themselves important, but measured in the presumption that changes in the surrogate reflect changes in an important outcome. The difference between desired and measured outcomes may relate to time frame. When there is a discrepancy between the time frame of measurement and that of interest, whether to rate down by one or two levels will depend on the magnitude of the discrepancy. Another source of indirectness related to measurement of outcomes is the use of substitute or surrogate endpoints in place of the exposed population’s important outcome of interest. In general, the use of a surrogate outcome requires rating down the quality of evidence by one, or even two, levels. Consideration of the biology, mechanism, and natural history of the disease can be helpful in making a decision about indirectness. Surrogates that are closer in the putative causal pathway to the adverse outcomes warrant rating down by only one level for indirectness. GRADE states that rarely, surrogates are sufficiently well established that one should choose not to rate down quality of evidence for indirectness. In general, evidence based on surrogate outcomes should usually trigger rating down, whereas the other types of indirectness will require a more considered judgment.

| **Rating for Indirectness**  0 no change  -1 decrease quality 1 level  -2 decrease quality 2 levels | | **Rationale for your judgment** |
| --- | --- | --- |
| Human |  |  |

**Category 3. Inconsistency of Evidence**

Possible ratings: 0 = no change; -1 or -2 downgrade 1 or 2 levels

According to Cochrane, “when studies yield widely differing estimates of effect (heterogeneity or variability in results) investigators should look for robust explanations for that heterogeneity.

…When heterogeneity exists and affects the interpretation of results, but authors fail to identify a plausible explanation, the quality of the evidence decreases.”

Based on GRADE, **a body of evidence is not rated up in quality if studies yield consistent results, but may be rated down in quality if inconsistent.** Their stated reason is that a consistent bias will lead to consistent, spurious findings.

GRADE suggests rating down the quality of evidence if large inconsistency (heterogeneity) in study results remains after exploration of a priori hypotheses that might explain heterogeneity. Judgment of the extent of heterogeneity is based on similarity of point estimates, extent of overlap of confidence intervals, and statistical criteria. GRADE’s recommendations refer to inconsistencies in effect size, specifically to relative measures (risk ratios and hazard ratios or odds ratios), not absolute measures.

Based on GRADE, reviewers should consider rating down for inconsistency when:

1. Point estimates vary widely across studies;
2. Confidence intervals (Andersonet al.) show minimal or no overlap;
3. The statistical test for heterogeneity-which tests the null hypothesis that all studies in a meta- analysis have the same underlying magnitude of effect- shows a low P-value;
4. The I*^2^* -which quantifies the proportion of the variation in point estimates due to among-study differences-is large. (I.e., the I^2^ index quantifies the degree of heterogeneity in a meta-analysis).

GRADE states that inconsistency is important only when it reduces confidence in results in relation to a particular decision. Even when inconsistency is large, it may not reduce confidence in results regarding a particular decision. For example, studies that are inconsistent related to the magnitude of a beneficial or harmful effect (but are in the same direction) would not be rated down; in instances when results are inconsistent as to whether there is a benefit or harm of treatment, GRADE would rate down the quality of evidence as a result of variability in results, because the meaning of the inconsistency is so relevant to the decision to treat or not to treat.

| **Rating for Inconsistency**  0 no change  -1 decrease quality 1 level  -2 decrease quality 2 levels | | **Rationale for your judgment** |
| --- | --- | --- |
| Human |  |  |

**Category 4. Imprecision of Evidence**

Possible ratings: 0=no change; -1 or -2 downgrade 1 or 2 levels

Cochrane states that when studies have few participants and few events, and thus have wide confidence intervals (Andersonet al. ), authors can lower their rating of the quality of evidence. These ratings of precision are made as judgments by review authors. The ratings are made by looking across studies, or, if available, on the results of a meta-analysis.

GRADE defines evidence quality differently for systematic reviews and guidelines. For systematic reviews, quality refers to confidence in the estimates of effect. For guidelines, quality refers to the extent to which confidence in the effect estimate is adequate to support a particular decision. For the purpose of step 3 of *Navigation Guide*, we will use the systematic review definition, because the decision phase does not occur until step 4 when recommendations for prevention are made. Thus, when reviewing the data for imprecision, evaluate your confidence in the estimate of the effect.

According to GRADE, to a large extent, CIs inform the impact of random error on evidence quality. Thus, when considering imprecision, the issue is whether the CI around the estimate of exposure effect is sufficiently narrow. If it is not, GRADE rates down the evidence quality by one level (for instance, from high to moderate). If the CI is very wide, GRADE might rate down by two levels.

| **Rating for Imprecision**  0 no change  -1 decrease quality 1 level  -2 decrease quality 2 levels | | **Rationale for your judgment** |
| --- | --- | --- |
| Human |  |  |

**Category 5. Publication Bias**

Possible ratings: 0 = no change; -1 or -2 downgrade 1 or 2 levels

GRADE and Cochrane assess publication bias in a similar manner. Whereas “selective outcome reporting” is assessed for each study included in the review as part of the risk of bias assessment, “publication bias” is assessed on the body of evidence. GRADE states that “when an entire study remains unreported and the results relate to the size of the effect- publication bias- one can assess the likelihood of publication bias only by looking at a group of studies.”

Cochrane’s definition of publication bias is “the *publication* or *non-publication* of research findings depending on the nature and direction of the results.” Cochrane and GRADE are primarily concerned with *overestimates* of true effects of treatments or pharmaceuticals, especially related to “small studies effects”, i.e., the tendency for estimates of an intervention to be more beneficial in smaller studies. There is empirical evidence in the clinical sciences that publication and other reporting biases result in over estimating the effects of interventions.

In contrast, in environmental health, we are primarily concerned with *underestimating* the true effects of a chemical exposure, since in many cases population wide exposure has already occurred. We are also concerned that studies finding no association are less likely to be published because journals are less likely to publish “negative” findings.

Applying this inverted concern to GRADE’s assessment for publication bias, leads to these considerations when rating publication bias:

- - Early *negative* studies, particularly if small in size, are suspect. (GRADE is concerned with early *positive* studies).
  - Authors of systematic reviews should suspect publication bias when studies are uniformly small, particularly when sponsored by the industry. (Same as GRADE)
  - Empirical examination of patterns of results (e.g., funnel plots) may suggest publication bias but should be interpreted with caution. (Same as GRADE)
  - More compelling than any of these theoretical exercises is authors’ success in obtaining the results of some unpublished studies and demonstrating that the published and unpublished data show different results. (Same as GRADE)
  - Comprehensive searches of the literature including unpublished studies, i.e., the grey literature, and a search for research in other languages are important to addressing publication bias. Note that Cochrane also states “comprehensive searching is not sufficient to prevent some substantial potential biases.”

| **Rating for Publication Bias**  0 no change  -1 decrease quality 1 level  -2 decrease quality 2 levels | | **Rationale for your judgment** |
| --- | --- | --- |
| Human |  |  |

**Upgrade Categories**

GRADE states that the circumstances for upgrading likely occur infrequently and are primarily relevant to observational and other non-randomized studies. Although it is possible to rate up results from randomized controlled trials, GRADE has yet to find a compelling circumstance for doing so. GRADE specifies 3 categories for increasing the quality of evidence.

**Category 6. Large Magnitude of Effect**

Possible ratings: 0 = no change; +1 or +2 upgrade 1 or 2 levels

Modelling studies suggests that confounding (from non-random allocation) alone is unlikely to explain associations with a relative risk (RR) greater than 2 (or less than 0.5), and very unlikely to explain associations with an RR greater than 5 (or less than 0.2). Thus, these are the definitions of “large magnitude of effect” used by GRADE to upgrade 1 or 2 levels, respectively. Also, GRADE is more likely to rate up if the effect is rapid and out of keeping with prior trajectory; usually supported by indirect evidence. GRADE presents empirical evidence to support these conclusions, and states that “although further research is warranted, both modelling and empirical work suggest the size of bias from confounding is unpredictable in direction but bounded in size.

Hence, the GRADE group has previously suggested guidelines for rating quality of evidence up by one category (typically from low to moderate) for associations greater than 2, and up by two categories for associations greater than 5.”

Applying the GRADE definitions of large magnitude of effect i.e., RR greater than 2 or 5 is problematic in environmental health because for dichotomous outcomes RR is a function of the exposure comparator; these definitions also are not applicable to results from continuous variables. At present, we do not have an empirically defined “large magnitude of effect.” Therefore, for the purpose of this case study, review authors should assess whether the results indicate a large magnitude of effect using their expert judgment of “large effects” in environmental health and state their definition for discussion by the group.

| **Rating for Large Magnitude of Effect**  0 no change  +1 increase quality 1 level  +2 increase quality 2 levels | | **Rationale for your judgment** |
| --- | --- | --- |
| Human |  |  |

**Category 7. Dose-response**

Possible ratings: 0 = no change; +1 or +2 upgrade 1 or 2 levels

Possible considerations include consistent dose response gradients in one or multiple studies, and/or dose response across studies, depending on the overall relevance to the body of evidence.

| **Rating for Dose-Response**  0 no change  +1 increase quality 1 level  +2 increase quality 2 levels | | **Rationale for your judgment** |
| --- | --- | --- |
| Human |  |  |

**Category 8. Residual Confounding Increases Confidence**

Possible ratings: 0=no change; +1 or +2 upgrade 1 or 2 levels

Upgrade if consideration of all plausible residual confounders, biases, or effect modification would underestimate the effect or suggest a spurious effect when results show no effect. If a study reports an association despite the presence of residual confounding, biases or effect modification that would diminish the association, confidence in the association is increased. GRADE provides an illustrative example related to bias: rating up observational evidence finding lack of association between vaccination and autism, which occurred despite empirically confirmed bias that parents of autistic children may be more likely to remember their vaccine experience. The negative findings despite this form of recall bias suggest rating up the quality of evidence.

| **Rating for Residual Confounding Increases Confidence**  0 no change  +1 increase quality 1 level  +2 increase quality 2 levels | | **Rationale for your judgment** |
| --- | --- | --- |
| Human |  |  |

The results of the reviewers’ ratings by population will be compiled and discussed leading to a final decision on overall quality of human evidence. The rationale for the decision will be fully documented.

**Final decision on overall quality of human evidence:**

(Example: Moderate quality is upgraded 1 step to high for XYZ reason(s))

---- High

---- Moderate

---- Low

# Appendix E: Rate the strength of evidence, Systematic Review 1

The strength of evidence for use as input data will be rated based on a combination of four criteria: (1) quality of the entire body of evidence; (2) population coverage of evidence (WHO regions and countries); (3) confidence in the entire body of evidence; and (4) other compelling attributes of the evidence that may influence certainty. The strength of evidence ratings are summarized below, where their meaning is further defined.

| Potentially sufficient evidence for use as input data | The body of evidence was rated as being of high or moderate quality; it covers at least one country each from at least two WHO regions; the review authors had confidence in the feasibility of using the evidence as input data; and there are no compelling attributes of the evidence that may reduce certainty in it. |
| --- | --- |
| Potentially insufficient or inadequate evidence for use as input data | The body of evidence was rated as of low quality; it covered fewer than one country each from at least two WHO regions; the review authors were very uncertain about the feasibility of using the evidence as input data or had no confidence in it; and/or there are compelling attributes of the evidence that may reduce certainty in it. |

# Appendix F: Overview of inclusion and exclusion criteria, Systematic Review 2

***Include***

1. Studies of working-age (≥15 year) workers in the formal or informal economy

2. Studies of workers residing in any Member States of WHO and/or ILO and working in any industrial setting or occupation

3. Studies that defined exposure to occupational ergonomic risk factors and osteoarthritis of hip or knee, and any selected other musculoskeletal disease respectively, in accordance with our standard definitions

4. Studies with the following designs: randomized controlled trials (including parallel-group, cluster, cross-over and factorial ones), prospective and retrospective cohort studies, case-control studies and other non-randomized intervention studies that estimate the effect of exposure to occupational ergonomic risk factors on osteoarthritis of hip or knee, and selected other musculoskeletal diseases respectively, for any years

5. Studies with measures of the relative effect of exposure to occupational ergonomic risk factors on the risk of having or developing osteoarthritis of hip or knee, and any selected other musculoskeletal diseases respectively, compared with the theoretical minimum risk exposure level

6. Included measures are relative effect measures, risk ratios and odds ratios for prevalence measures and hazard ratios for incidence measures

7. Studies published in any year and any language

***Exclude***

1. Studies of unpaid domestic workers

2. Studies of children (aged ˂15 years)

3. Studies reporting on measures of absolute effects (e.g. mean differences in risks or odds), if they cannot be converted into eligible relative measures

4. Cross-sectional studies, qualitative, modelling and case studies, as well as non-original studies without quantitative data (e.g. letters, commentaries and perspectives)

# Appendix G: Proposed Ovid Medline search strategy, Systematic Review 2

| **ELBOW** |  |  |  |  |
| --- | --- | --- | --- | --- |
|  |  |  |  |  |
|  |  | **Ovid MEDLINE(R) Epub Ahead of Print, In-Process & Other Non-Indexed Citations, Ovid MEDLINE(R) Daily and Ovid MEDLINE(R) <1946 to Present>** | | |
|  | **#** | **Searches** |  |  |
|  | 1 | elbow/ or hand arm vibration syndrome/ or exp cumulative trauma disorders/ or exp arm injuries/ |  |  |
|  | 2 | (elbow? or medial epicondylitis or Lateral Epicondylitis or Olecranon bursitis).ab,kf,ti. |  |  |
|  | 3 | or/1-2 [anatomy \| relevant diseases] |  |  |
|  | 4 | exp Occupations/ or occupational groups/ or Workload/ or exp Work/ or Workplace/ or exp Occupational Diseases/ or Rehabilitation, Vocational/ or Occupational Health/ or Sick Leave/ or Absenteeism/ or Retirement/ or workers' compensation/ or exp Employment/ or exp Occupational Exposure/ or Volunteers/ or exp industrial sector/ |  |  |
|  | 5 | (worka* or worke* or workg* or worki* or workl* or workp* or work capacity or work disabilit* or work abilit* or at work or work exposure or work related or workers or job* or employee or staff or personnel or occupation or occupations or occupational or outdoor work* or day shift* or night shift* or shift work* or vocational rehabilitation or sick leave or absenteeism or sickness absen* or absente* or presente* or "return to work" or vocational reintegration or retirement or pension or employment or unemployed or unemployment or work status or industries or industrial sector or volunteer* or voluntary worker* or repetitive work).ab,kf,ti. |  |  |
|  | 6 | or/4-5 |  |  |
|  | 7 | posture/ or evaluation studies as topic/ or exp causality/ or risk assessment/ or time factors/ or lifting/ or vibration/ or weight bearing/ or cumulative trauma disorders/ |  |  |
|  | 8 | ((posture adj3 wrist?) or (elevat* adj3 arm?) or "computer use" or "compuater usage" or kneel* or climbing or repetition strain or repetitive strain or repetitive motion or repetitive movement? or (repetitive adj5 activit*) or repetition motion or overuse or biomechanical or lift* or manual work* or manual labo* or carry* or elevate? or arm force or hand force or hand vibration or arm vibration or (Handling adj2 (heavy or load? or weight)) or Handgrip force or Vibrating tool? or Handling tools or risk? or course? or etiolog* or causality or exposure?).ab,kf,ti. |  |  |
|  | 9 | evaluation studies.pt. |  |  |
|  | 10 | etiology.fs. |  |  |
|  | 11 | or/7-10 [etiology \| (known) risk] |  |  |
|  | 12 | 3 and 6 and 11 |  |  |
|  | 13 | animals/ not humans/ |  |  |
|  | 14 | 12 not 13 |  |  |
|  | 15 | 13 not 14 |  |  |
|  | 16 | remove duplicates from 14 |  |  |
|  | 17 | remove duplicates from 15 |  |  |
|  | 18 | 16 or 17 |  |  |
|  |  |  |  |  |
|  |  |  |  |  |
| **KNEE** |  |  |  |  |
|  |  |  |  |  |
|  |  | **Ovid MEDLINE(R) Epub Ahead of Print, In-Process & Other Non-Indexed Citations, Ovid MEDLINE(R) Daily and Ovid MEDLINE(R) <1946 to Present>** | | |
|  | **#** | **Searches** |  |  |
|  | 1 | knee/ or knee joint/ or "menisci, tibial"/ or exp cumulative trauma disorders/ or chondromalacia patellae/ or exp knee injuries/ or tibial meniscus injuries/ |  |  |
|  | 2 | (knee? or Superior tibiofibular joint or proximal tibiofibular articulation or Prepatellar bursitis or chondromalacia patella* or lateral menisc* or tibial menisc* or medial meniscus or meniscus injur* or meniscus disorder?).ab,kf,ti. |  |  |
|  | 3 | or/1-2 [anatomy \| relevant diseases] |  |  |
|  | 4 | exp Occupations/ or exp occupational groups/ or Workload/ or exp Work/ or Workplace/ or exp Occupational Diseases/ or Rehabilitation, Vocational/ or Occupational Health/ or Sick Leave/ or Absenteeism/ or Retirement/ or workers' compensation/ or exp Employment/ or exp Occupational Exposure/ or Volunteers/ or exp industrial sector/ |  |  |
|  | 5 | (worka* or worke* or workg* or worki* or workl* or workp* or work capacity or work disabilit* or work abilit* or at work or work exposure or work related or workers or job* or employee or staff or personnel or occupation or occupations or occupational or outdoor work* or day shift* or night shift* or shift work* or vocational rehabilitation or sick leave or absenteeism or sickness absen* or absente* or presente* or "return to work" or vocational reintegration or retirement or pension or employment or unemployed or unemployment or work status or industries or industrial sector or volunteer* or voluntary worker* or repetitive work).ab,kf,ti. |  |  |
|  | 6 | or/4-5 [work] |  |  |
|  | 7 | evaluation studies as topic/ or exp causality/ or risk assessment/ or time factors/ or lifting/ or weight bearing/ or cumulative trauma disorders/ |  |  |
|  | 8 | (kneel* or squatting or climbing or physcial work or repetition strain or repetitive strain or repetitive motion or repetition motion or (repetitive adj5 activit*) or overuse or biomechanical or lift* or manual work* or manual labo* or carry* or elevate? or risk? or course? or etiolog* or causality or exposure?).ab,kf,ti. |  |  |
|  | 9 | evaluation studies.pt. |  |  |
|  | 10 | etiology.fs. |  |  |
|  | 11 | or/7-10 [etiology \| (known) risk] |  |  |
|  | 12 | 3 and 6 and 11 |  |  |
|  | 13 | animals/ not humans/ |  |  |
|  | 14 | 12 not 13 |  |  |
|  | 15 | 13 not 14 |  |  |
|  | 16 | remove duplicates from 14 |  |  |
|  | 17 | remove duplicates from 15 |  |  |
|  | 18 | 16 or 17 |  |  |
|  |  |  |  |  |
|  |  |  |  |  |
| **SHOULDER** | |  |  |  |
|  |  | **Ovid MEDLINE(R) Epub Ahead of Print, In-Process & Other Non-Indexed Citations, Ovid MEDLINE(R) Daily and Ovid MEDLINE(R) <1946 to Present>** | | |
|  | 1 | shoulder joint/ or rotator cuff/ or shoulder pain/ or shoulder impingement syndrome/ or hand arm vibration syndrome/ or exp cumulative trauma disorders/ |  |  |
|  | 2 | (((arm? or shoulder? or upper limb? or upper extremit*) adj3 (vibration or repetitive)) or shoulder pain or (shoulder? adj5 (complaint? or disorder? or extertion or flexion or repetitive)) or shoulder impingement or rotator cuff syndrome or cumulative trauma disorder?).ab,kf,ti |  |  |
|  | 3 | or/1-2 |  |  |
|  | 4 | exp Occupations/ or Workload/ or exp Work/ or Workplace/ or exp Occupational Diseases/ or Rehabilitation, Vocational/ or Occupational Health/ or Sick Leave/ or Absenteeism/ or Retirement/ or workers' compensation/ or exp Employment/ or exp Occupational Exposure/ or Volunteers/ or exp industrial sector/ |  |  |
|  | 5 | (worka* or worke* or workg* or worki* or workl* or workp* or work capacity or work disabilit* or work abilit* or at work or work exposure or work related or workers or job* or employee or staff or personnel or occupation or occupations or occupational or outdoor work* or day shift* or night shift* or shift work* or vocational rehabilitation or sick leave or absenteeism or sickness absen* or absente* or presente* or "return to work" or vocational reintegration or retirement or pension or employment or unemployed or unemployment or work status or industries or industrial sector or volunteer* or voluntary worker* or repetitive work).ab,kf,ti. |  |  |
|  | 6 | or/4-5 |  |  |
|  | 7 | posture/ or evaluation studies as topic/ or exp causality/ or risk assessment/ or time factors/ or lifting/ or vibration/ or weight bearing/ or exp age factors/ |  |  |
|  | 8 | (lift* or carry* or elevate? or work above or risk? or course? or etiolog* or causality or exposure?).ab,kf,ti |  |  |
|  | 9 | evaluation studies.pt |  |  |
|  | 10 | etiology.fs |  |  |
|  | 11 | or/7-10 |  |  |
|  | 12 | 3 and 6 and 11 |  |  |
|  | 13 | animals/ not humans/ |  |  |
|  | 14 | 12 not 13 |  |  |
|  | 15 | ..dedup 14 |  |  |
|  |  |  |  |  |
|  |  | **Ovid Embase** |  |  |
|  | 1 | *shoulder/ or exp *rotator cuff/ or *shoulder pain/ or *shoulder impingement syndrome/ or *hand arm vibration/ or exp *cumulative trauma disorder/ |  |  |
|  | 2 | (((arm? or shoulder? or upper limb? or upper extremit*) adj3 (vibration or repetitive)) or shoulder pain or (shoulder? adj5 (complaint? or disorder? or extertion or flexion or repetitive)) or shoulder impingement or rotator cuff syndrome or cumulative trauma disorder?).ab,kw,ti |  |  |
|  | 3 | or/1-2 |  |  |
|  | 4 | exp Occupation/ or exp Work/ or exp Occupational Disease/ or exp Occupational exposure/ or workman compensation/ or Volunteer/ or industrial sector/ |  |  |
|  | 5 | (worka* or worke* or workg* or worki* or workl* or workp* or work capacity or work disabilit* or work abilit* or at work or work exposure or work related or workers or job* or employee or staff or personnel or occupation or occupations or occupational or outdoor work* or day shift* or night shift* or shift work* or vocational rehabilitation or sick leave or absenteeism or sickness absen* or absente* or presente* or "return to work" or vocational reintegration or retirement or pension or employment or unemployed or unemployment or work status or industries or industrial sector or volunteer* or voluntary worker* or repetitive work).ab,kw,ti. |  |  |
|  | 6 | Occupational Health and Industrial Medicine .ec. |  |  |
|  | 7 | or/4-6 |  |  |
|  | 8 | body posture/ or evaluation study/ or risk assessment/ or time/ or biomechanics/ or vibration/ or weight bearing/ or etiology/ |  |  |
|  | 9 | (lift* or carry* or elevate? or work above or risk? or course? or etiolog* or causality or exposure?).ab,kw,ti |  |  |
|  | 10 | et.fs |  |  |
|  | 11 | or/8-10 |  |  |
|  | 12 | 3 and 7 and 11 |  |  |
|  | 13 | (animal/ or animal experiment/ or animal model/ or nonhuman/ or rat/ or mouse/ or (rat or rats or mouse or mice).ti.) not human/ |  |  |
|  | 14 | 12 not 13 |  |  |
|  | 15 | ..dedup 14 |  |  |
|  |  |  |  |  |
| **HIP** |  |  |  |  |
|  |  | **Ovid MEDLINE(R) Epub Ahead of Print, In-Process & Other Non-Indexed Citations, Ovid MEDLINE(R) Daily and Ovid MEDLINE(R) <1946 to Present>** | | |
|  | 1 | Osteoarthritis, Hip/ or (hip/ and osteoarthritis/) |  |  |
|  | 2 | coxarthr* or ((osteoarthr* or arthr* or oa) adj2 (cox? or hip?)) or (joint disease adj3 (cox? or hip?)).ab,kf,ti |  |  |
|  | 3 | or/1-2 |  |  |
|  | 4 | exp Occupations/ or Workload/ or exp Work/ or Workplace/ or exp Occupational Diseases/ or Rehabilitation, Vocational/ or Occupational Health/ or Sick Leave/ or Absenteeism/ or Retirement/ or workers' compensation/ or exp Employment/ or exp Occupational Exposure/ or Volunteers/ or exp industrial sector/ |  |  |
|  | 5 | (worka* or worke* or workg* or worki* or workl* or workp* or work capacity or work disabilit* or work abilit* or at work or work exposure or work related or workers or job* or employee or staff or personnel or occupation or occupations or occupational or outdoor work* or day shift* or night shift* or shift work* or vocational rehabilitation or sick leave or absenteeism or sickness absen* or absente* or presente* or "return to work" or vocational reintegration or retirement or pension or employment or unemployed or unemployment or work status or industries or industrial sector or volunteer* or voluntary worker* or repetitive work).ab,kf,ti. |  |  |
|  | 6 | or/4-5 |  |  |
|  | 7 | evaluation studies as topic/ or exp causality/ or risk assessment/ or time factors/ or lifting/ or weight bearing/ or workload/ |  |  |
|  | 8 | (lift* or carry* or workload? or work load? or risk? or course? or etiolog* or causality or exposure?).ab,kf,ti |  |  |
|  | 9 | evaluation studies.pt |  |  |
|  | 10 | etiology.fs |  |  |
|  | 11 | or/7-10 [etiology \| (known) risk] |  |  |
|  | 12 | 3 and 6 and 11 |  |  |
|  | 13 | animals/ not humans/ |  |  |
|  | 14 | 12 not 13 |  |  |
|  | 15 | ..dedup 14 |  |  |

# Appendix H: Rate risk of bias, Systematic Review 2

Most of the text from these instructions and criteria for judging risk of bias has been adopted verbatim or adapted from one of the latest *Navigation Guide* (Woodruff and Sutton 2014) systematic reviews (Lamet al. 2016).

Instructions:

- - *Please evaluate each individual study for the following nine risk of bias domains. Please answer “low risk,” “probably low risk,” “probably high risk,” “high risk,” or “not applicable” and provide details/justification for your rating. If there is empirical evidence or other knowledge that informs the direction of bias, please include this in your answer as well; however, if there is not enough information to do so please do not guess at the direction of bias.*
  - *Additionally, please note that some internal validity issues could potentially be appropriately captured in several different risk of bias considerations. In this situation, please select the single most appropriate domain to evaluate this potential bias, to avoid double-counting the same internal validity concern.*

1. **Are the study groups at risk of not representing their source populations in a manner that might introduce selection bias?**

The source population is viewed as the population for which study investigators are targeting their study question of interest. Examples of considerations for this risk of bias domain include: 1) level of detail reported for participant inclusion/exclusion (including details from previously published papers referenced in the article for an existing cohort); 2) participation rates and whether this differed by exposure or outcome group; 3) attrition rates and reasons; and 4) comparisons of study characteristics between the study population and full cohort.

Criteria for a judgment of LOW risk of bias (i.e. answer: “No”): EITHER:

- 1. The descriptions of the source population, inclusion/exclusion criteria, recruitment and enrollment procedures, participation and follow-up rates were sufficiently detailed, and adequate data were supplied on the distribution of relevant study sample and population characteristics to support the assertion that risk of selection effects was minimal.

OR

- 1. Although the descriptions and/or data as indicated in “a” above suggested the potential for selection effects, adequate support was given indicating that potential selection effects were *not* differential across both exposure and outcome.

OR

- 1. Although the descriptions and/or data as indicated in “a” above suggested the potential for selection effects and there was no support indicating that potential selection effects were *not* differential across both exposure and outcome, selection factors appeared to be well-understood, were measured in the data set, and appropriate adjustment post hoc techniques were used to control for selection bias.

Criteria for the judgment of PROBABLY LOW risk of bias (i.e. answer: “Probably No”):

There is insufficient information about participant selection to permit a judgment of low risk of bias, but there is indirect evidence which suggests that inclusion/exclusion criteria, recruitment and enrollment procedures, and participation and follow-up rates were consistent across groups as described by the criteria for a judgment of low risk of bias.

Criteria for the judgment of PROBABLY HIGH risk of bias (i.e. answer: “Probably Yes”):

There is insufficient information about participant selection to permit a judgment of high risk of bias, but there is indirect evidence which suggests that inclusion/exclusion criteria, recruitment and enrollment procedures, and participation and follow-up rates were inconsistent across groups, as described by the criteria for a judgment of high risk of bias.

Criteria for the judgment of HIGH risk of bias (i.e. answer: “Yes”):

1. There were indications from descriptions of the source population, inclusion/exclusion criteria, recruitment and enrolment procedures, participation and follow-up rates, or data on the distribution of relevant study sample and population characteristics that risk of selection effects were substantial; and
2. There was no support to indicate that potential selection effects were *not* differential across both exposure and outcome; and
3. Adjustment post hoc techniques were not used to control for selection bias.

Criteria for the judgment of NOT APPLICABLE (risk of bias domain is not applicable to study):

There is evidence that participant selection is not an element of study design capable of introducing risk of bias in the study.

1. **Was knowledge of the group assignments inadequately prevented (i.e. blinded or masked) during the study, potentially leading to subjective measurement of either exposure or outcome?**

Criteria for a judgment of LOW risk of bias (i.e. answer: “No”): Any of the following:

- No blinding, but the review authors judge that the outcome measures as well as the exposure measures are not likely to be influenced by lack of blinding (such as differential outcome assessment where the outcome is assessed using different measurement or estimation metrics across the exposure groups, or differential exposure assessment where exposure is assessed using different measurement or estimation metrics across the diagnostic or outcome groups); or
- Blinding of key study personnel was ensured, and it is unlikely that the blinding could have been broken; or
- Some key study personnel were not blinded, but exposure and outcome assessment was blinded and the non-blinding of others is unlikely to introduce bias.

Criteria for the judgment of PROBABLY LOW risk of bias (i.e. answer: “Probably No”):

There is insufficient information about blinding to permit a judgment of low risk of bias, but there is indirect evidence which suggests the study was adequately blinded, as described by the criteria for a judgment of low risk of bias. For example, investigators were effectively blinded to the exposure and/or outcome groups if the exposure was measured by a separate entity and the outcome was obtained from a hospital record.

Criteria for the judgment of PROBABLY HIGH risk of bias (i.e. answer: “Probably Yes”):

There is insufficient information about blinding to permit a judgment of high risk of bias, but there is indirect evidence which suggests the study was not adequately blinded, as described by the criteria for a judgment of high risk of bias.

Criteria for the judgment of HIGH risk of bias (i.e. answer: “Yes”): Any of the following:

- No blinding or incomplete blinding, and the outcome measures or exposure measures is likely to be influenced by lack of blinding (i.e. differential outcome or exposure assessment); or
- Blinding of key study personnel attempted, but likely that the blinding could have been broken so as to introduce bias; or
- Some key study personnel were not blinded, and the non-blinding of others was likely to introduce bias.

Criteria for the judgment of NOT APPLICABLE (risk of bias domain is not applicable to study):

There is evidence that blinding is not an element of study design capable of introducing risk of bias in the study.

1. **Were exposure assessment methods lacking accuracy?**

*The following list of considerations represents a collection of factors proposed by experts in various fields that may potentially influence the internal validity of the exposure assessment in a systematic manner (not those that may randomly affect overall study results).* ***These should be interpreted only as suggested considerations, and should not be viewed as scoring or a checklist.***

**List of Considerations**:

*Possible sources of exposure assessment metrics:*

1. *Official Records (Ministry of Health, Ministry of Labour or other official sources)*
2. *Organization*
3. *Self-reported*
4. *Combination of the above options*

*For each, overall considerations include:*

1. *What is the quality of the source of the metric being used?*
2. *Is the exposure measured in the study a surrogate for the exposure?*
3. *What was the temporal coverage (i.e. short or long-term exposure)?*
4. *Did the analysis account for prediction uncertainty?*
5. *How was missing data accounted for, and any data imputations incorporated?*
6. *Were sensitivity analyses performed?*

*In particular, for exposure assessment models:*

1. *Were the input data in the study suspected to systematically under- or over-estimate exposure?*
2. *What type of model was used?*
3. *What was geographic/spatial accuracy (county, census tract, organization, individual residence)?*
4. *What was the temporal specificity and variation?*
5. *What was the space-time coverage of the model?*
6. *Were time-activity patterns accounted for?*

Criteria for a judgment of LOW risk of bias (i.e. answer: “No”):

The reviewers judge that there is low risk of exposure misclassification, i.e.:

- - There is high confidence in the accuracy of the exposure assessment methods, such as methods that have been tested for validity and reliability in measuring the targeted exposure.

Criteria for the judgment of PROBABLY LOW risk of bias (i.e. answer: “Probably No”):

There is insufficient information about the exposure assessment methods to permit a judgment of low risk of bias, but there is indirect evidence that suggests that methods were robust, as described by the criteria for a judgment of low risk of bias.

Criteria for the judgment of PROBABLY HIGH risk of bias (i.e. answer: “Probably Yes”):

There is insufficient information about the exposure assessment methods to permit a judgment of high risk of bias, but there is indirect evidence that suggests that methods were not robust, as described by the criteria for a judgment of high risk of bias.

Criteria for the judgment of HIGH risk of bias (i.e. answer: “Yes”):

The reviewers judge that there is high risk of exposure misclassification and any one of the following:

- - - There is low confidence in the accuracy of the exposure assessment methods; or
    - Less-established or less direct exposure measurements are not validated and are suspected to introduce bias that impacts the outcome assessment; or
    - Uncertain how exposure information was obtained.

Criteria for the judgment of NOT APPLICABLE (risk of bias domain is not applicable to study):

There is evidence that exposure assessment methods are not capable of introducing risk of bias in the study.

1. **Were outcome assessment methods lacking accuracy?**

Criteria for a judgment of LOW risk of bias (i.e. answer: “No”):

The reviewers judge that there is low risk of outcome misclassification, i.e.:

- Outcomes were assessed and defined consistently across all study participants, using valid and reliable measures (all cases of osteoarthritis of hip or knee and selected other musculoskeletal diseases with solid medical records). Note that all outcome assessment measures captured in the PECO statement are considered beforehand to be valid and reliable, unless other information provided within the study warrants a consideration otherwise; or
- Less-established or less direct outcome measurements are validated against well- established or direct methods; or
- Appropriate sensitivity analyses were conducted that suggest the influence of outcome misclassification would be minimal
- AND, if applicable, appropriate QA/QC for methods is described and is satisfactory.

Criteria for the judgment of PROBABLY LOW risk of bias (i.e. answer: “Probably No”):

There is insufficient information about the outcome assessment methods to permit a judgment of low risk of bias, but there is indirect evidence which suggests that methods were robust, as described by the criteria for a judgment of low risk of bias. Appropriate QA/QC for methods are not described but the review authors judge that the outcome and the outcome assessment are objective and uniform across study groups.

Criteria for the judgment of PROBABLY HIGH risk of bias (i.e. answer: “Probably Yes”):

There is insufficient information about the outcome assessment methods to permit a judgment of high risk of bias, but there is indirect evidence which suggests that methods were not robust, as described by the criteria for a judgment of high risk of bias.

Criteria for the judgment of HIGH risk of bias (i.e. answer: “Yes”):

The reviewers judge that there is high risk of outcome misclassification and any one of the following:

- There is low confidence in the accuracy of the outcome assessment methods; or
- Less-established or less direct outcome measurements are not validated and are suspected to introduce bias that impacts the outcome assessment
- Uncertain how outcome information was obtained

Criteria for the judgment of NOT APPLICABLE (risk of bias domain is not applicable to study):

There is evidence that outcome assessment methods are not capable of introducing risk of bias in the study.

1. **Was potential confounding inadequately incorporated?**

List of important potential confounders, collectively generated by review authors prior to the initiation of screening for studies based on expert opinion and knowledge gathered from the literature:

Tier I: Important confounders:

- Age, sex, socioeconomic position, body mass index, smoking status and sporting and/or leisure activities.

Tier II: Other potentially important confounders:

- None identified.

Criteria for a judgment of LOW risk of bias (i.e. answer: “No”):

- The study appropriately assessed and accounted for (i.e. matched, stratified, excluded certain populations or statistically controlled for) all important confounders (Tier I) using appropriate statistical techniques, or reported that important confounders were evaluated and omitted because inclusion did not substantially affect the results. The determination of specific confounders may also be informed by, but not limited to, the studies included in the overall review,
- AND the study appropriately assessed and accounted for (i.e. matched, stratified, or statistically controlled for) other potentially important confounders relevant (Tier II) using appropriate statistical techniques, or reported that these confounders were evaluated and omitted because inclusion did not substantially affect the results,
- AND the important potential confounders were measured consistently across study groups using valid and reliable methods, or the influence of covariate measurement error was determined, through sensitivity analysis, to be minimal.

Criteria for the judgment of PROBABLY LOW risk of bias (i.e. answer: “Probably No”):

- The study appropriately accounted for most but not all of the important confounders (Tier I) or used appropriate statistical techniques;
- AND some of the other potentially important confounders relevant (Tier II) using appropriate statistical techniques,
- OR reported that these confounders were evaluated and omitted because inclusion did not substantially affect the results;
- AND this is not expected to introduce substantial bias.

Criteria for the judgment of PROBABLY HIGH risk of bias (i.e. answer: “Probably Yes”):

- The study evaluated some but not all of the important confounders (Tier I),
- AND some but not all of the other potentially important confounders relevant (Tier II), OR used questionable statistical techniques for confounder adjustment;
- AND this is expected to introduce substantial bias.

Criteria for the judgment of HIGH risk of bias (i.e. answer: “Yes”):

- The study did not account for or evaluate multiple important confounders (Tier I),
- AND did not account for or evaluate multiple other potentially important confounders relevant (Tier II),
- OR the important potential confounders were inappropriately measured and/or inappropriately analyzed across study groups.

Criteria for the judgment of NOT APPLICABLE (risk of bias domain is not applicable to study):

There is evidence that outcome assessment methods are not capable of introducing risk of bias in the study.

1. **Were incomplete outcome data inadequately addressed?**

Criteria for a judgment of LOW risk of bias (i.e. answer: “No”):

Participants were followed long enough to obtain outcome measurements

OR any one of the following:

- No missing outcome data; or
- Reasons for missing outcome data unlikely to be related to true outcome (for survival data, censoring unlikely to introduce bias); or
- Attrition or missing outcome data balanced in numbers across exposure groups, with similar reasons for missing data across groups; or
- For dichotomous outcome data, the proportion of missing outcomes compared with observed event risk not enough to have a relevant impact on the exposure effect estimate; or
- For continuous outcome data, plausible effect size (difference in means or standardized difference in means) among missing outcomes not enough to have a relevant impact on the observed effect size; or
- Missing data have been imputed using appropriate methods

Criteria for the judgment of PROBABLY LOW risk of bias (i.e. answer: “Probably No”):

There is insufficient information about incomplete outcome data to permit a judgment of low risk of bias, but there is indirect evidence which suggests incomplete outcome data was adequately addressed, as described by the criteria for a judgment of low risk of bias.

Criteria for the judgment of PROBABLY HIGH risk of bias (i.e. answer: “Probably Yes”):

There is insufficient information about incomplete outcome data to permit a judgment of high risk of bias, but there is indirect evidence which suggests incomplete outcome data was not adequately addressed, as described by the criteria for a judgment of high risk of bias.

Criteria for the judgment of HIGH risk of bias (i.e. answer: “Yes”):

Participants were not followed long enough to obtain outcome measurements OR any one of the following:

- Reason for missing outcome data likely to be related to true outcome, with either imbalance in numbers or reasons for missing data across exposure groups; or
- For dichotomous outcome data, the proportion of missing outcomes compared with observed event risk enough to induce biologically relevant bias in intervention effect estimate; or
- For continuous outcome data, plausible effect size (difference in means or standardized difference in means) among missing outcomes enough to induce biologically relevant bias in observed effect size; or
- Potentially inappropriate application of imputation.

Criteria for the judgment of NOT APPLICABLE (risk of bias domain is not applicable to study):

There is evidence that incomplete outcome data is not capable of introducing risk of bias in the study.

1. **Does the study report appear to have selective outcome reporting?**

Criteria for a judgment of LOW risk of bias (i.e. answer: “No”):

All of the study’s pre-specified (primary and secondary) outcomes outlined in the pre- published protocol or the published manuscript’s methods, abstract, and/or introduction section that are of interest in the review have been reported in the pre-specified way.

Criteria for the judgment of PROBABLY LOW risk of bias (i.e. answer: “Probably No”):

There is insufficient information about selective outcome reporting to permit a judgment of low risk of bias, but there is indirect evidence which suggests the study was free of selective reporting, as described by the criteria for a judgment of low risk of bias. This includes if a pre-published protocol is not available but the study’s pre-specified (primary and secondary) outcomes outlined in the published manuscript’s methods, abstract, and/or introduction section that are of interest in the review have been reported in the pre-specified way.

Criteria for the judgment of PROBABLY HIGH risk of bias (i.e. answer: “Probably Yes”):

There is insufficient information about selective outcome reporting to permit a judgment of high risk of bias, but there is indirect evidence which suggests the study was not free of selective reporting, as described by the criteria for a judgment of high risk of bias. This includes if a pre-published protocol is not available and the study’s pre-specified (primary and secondary) outcomes outlined in the published manuscript’s methods, abstract, and/or introduction section that are of interest in the review have not been reported in the pre-specified way.

Criteria for the judgment of HIGH risk of bias (i.e. answer: “Yes”): Any one of the following:

- Not all of the study’s pre-specified primary outcomes (as outlined in the pre-published protocol or published manuscript’s methods, abstract, and/or introduction) have been reported; or
- One or more primary outcomes is reported using measurements, analysis methods or subsets of the data (e.g. subscales) that were not pre-specified; or
- One or more reported primary outcomes were not pre-specified (unless clear justification for their reporting is provided, such as an unexpected effect); or
- One or more outcomes of interest are reported incompletely

Criteria for the judgment of NOT APPLICABLE (risk of bias domain is not applicable to study):

There is evidence that selective outcome reporting is not capable of introducing risk of bias in the study.

1. **Did the study receive any support from a company, study author, or other entity having a financial interest in any of the exposures studied?**

Criteria for a judgment of LOW risk of bias (i.e. answer: “No”):

The study did not receive support from a company, study author, or other entity having a financial interest in the outcome of the study. Examples include the following:

- Funding source is limited to government, non-profit organizations, or academic grants funded by government, foundations and/or non-profit organizations;
- Chemicals or other treatment used in study were purchased from a supplier;
- Company affiliated staff are not mentioned in the acknowledgements section;
- Authors were not employees of a company with a financial interest in the outcome of the study;
- Company with a financial interest in the outcome of the study was not involved in the design, conduct, analysis, or reporting of the study and authors had complete access to the data;
- Study authors make a claim denying conflicts of interest;
- Study authors are unaffiliated with companies with financial interest, and there is no reason to believe a conflict of interest exists;
- All study authors are affiliated with a government agency (are prohibited from involvement in projects for which there is a conflict of interest or an appearance of conflict of interest).

Criteria for the judgment of PROBABLY LOW risk of bias (i.e. answer: “Probably No”):

There is insufficient information to permit a judgment of low risk of bias, but there is indirect evidence which suggests the study was free of support from a company, study author, or other entity having a financial interest in the outcome of the study, as described by the criteria for a judgment of low risk of bias.

Criteria for the judgment of PROBABLY HIGH risk of bias (i.e. answer: “Probably Yes”):

There is insufficient information to permit a judgment of high risk of bias, but there is indirect evidence which suggests the study was not free of support from a company, study author, or other entity having a financial interest in the outcome of the study, as described by the criteria for a judgment of high risk of bias.

Criteria for the judgment of HIGH risk of bias (i.e. answer: “Yes”):

The study received support from a company, study author, or other entity having a financial interest in the outcome of the study. Examples of support include:

- Research funds;
- Chemicals, equipment or testing provided at no cost;
- Writing services;
- Author/staff from study was employee or otherwise affiliated with company with financial interest;
- Company limited author access to the data;
- Company was involved in the design, conduct, analysis, or reporting of the study;
- Study authors claim a conflict of interest

Criteria for the judgment of NOT APPLICABLE (risk of bias domain is not applicable to study):

There is evidence that conflicts of interest are not capable of introducing risk of bias in the study.

1. **Did the study appear to have other problems that could put it at a risk of bias?**

Criteria for a judgment of LOW risk of bias (i.e. answer: “No”):

The study appears to be free of other sources of bias.

Criteria for the judgment of PROBABLY LOW risk of bias (i.e. answer: “Probably No”):

There is insufficient information to permit a judgment of low risk of bias, but there is indirect evidence which suggests the study was free of other threats to validity.

Criteria for the judgment of PROBABLY HIGH risk of bias (i.e. answer: “Probably Yes”):

There is insufficient information to permit a judgment of high risk of bias, but there is indirect evidence which suggests the study was not free of other threats to validity, as described by the criteria for a judgment of high risk of bias.

Criteria for the judgment of HIGH risk of bias (i.e. answer: “Yes”):

There is at least one important risk of bias. For example, the study:

- Had a potential source of bias related to the specific study design used; or
- Stopped early due to some data-dependent process (including a formal-stopping rule); or
- The conduct of the study is affected by interim results (e.g. recruiting additional participants from a subgroup showing greater or lesser effect); or
- Has been claimed to have been fraudulent; or
- Had some other problem.

# Appendix I: Rate the strength of evidence, Systematic Review 2

The strength of evidence will be rated based on a combination of four criteria: (1) Quality of the entire body of evidence; (2) Direction of the effect estimate; (3) Confidence in the effect estimate; and (4) Other compelling attributes of the evidence that may influence certainty. The strength of evidence ratings are summarized below, where their meaning is further defined.

| Sufficient evidence of toxicity/harmfulness | The available evidence usually includes consistent results from well‐designed, well‐conducted studies, and the conclusion is unlikely to be strongly affected by the results of future studies. For human evidence a positive relationship is observed between exposure and outcome where chance, bias, and confounding, can be ruled out with reasonable confidence. |
| --- | --- |
| Limited evidence of toxicity/harmfulness | The available evidence is sufficient to determine the effects of the exposure, but confidence in the estimate is constrained by such factors as: the number, size, or quality of individual studies, the confidence in the effect, or inconsistency of findings across individual studies. As more information becomes available, the observed effect could change, and this change may be large enough to alter the conclusion. For human evidence a positive relationship is observed between exposure and outcome where chance, bias, and confounding cannot be ruled out with reasonable confidence. |
| Inadequate evidence of toxicity/harmfulness | Studies permit no conclusion about a toxic effect. The available evidence is insufficient to assess effects of the exposure. Evidence is insufficient because of: the limited number or size of studies, low quality of individual studies, or inconsistency of findings across individual studies. More information may allow an estimation of effects. |
| Evidence of lack of toxicity/harmfulness | The available evidence includes consistent results from well‐designed, well‐conducted studies, and the conclusion is unlikely to be strongly affected by the results of future studies. For human evidence more than one study showed no effect on the outcome of interest at the full range of exposure levels that humans are known to encounter, where bias and confounding can be ruled out with reasonable confidence. The conclusion is limited to the age at exposure and/or other conditions and levels of exposure studied. |

# Reference list for all appendices

Anderson, L.M.; Petticrew, M.; Rehfuess, E.; Armstrong, R.; Ueffing, E.; Baker, P.; Francis, D.; Tugwell, P. Using logic models to capture complexity in systematic reviews. Research synthesis methods 2011;2:33-42

Descatha, Alexis.; Grace Sembajwe, Michel Baer, Fabio Boccuni, Cristina Di Tecco, Clément Duret, Bradley A Evanoff, Diana Gagliardi, Ivan D Ivanov, Nancy Leppink, Linda L Magnusson Hanson, Alessandro Marinaccio, Anna Ozguler, Frank Pega, Fernando Pico, Annette M Prüss-Üstün, Matteo Ronchetti, Yves Roquelaure, Erika Sabbath, Gretchen A Stevens, Akizumi Tsutsumi, Yuka Ujita, and Sergio Iavicoli. 2018. WHO/ILO work-related burden of disease and injury: Protocol for systematic reviews of exposure to long working hours and of the effect of exposure to long working hours on stroke. Environment International 2018;19:366-378

Godderis, Lode, Jelena Bakusic, Emma Boonen, Ellen Delvaux, Ivan D Ivanov, Marie-Claire Lambrechts, Carolina OC Latorraca, Nancy Leppink, Ana LC Martimbianco, Frank Pega, Annette M Prüss-Üstün, Rachel Riera, Yuka Ujita, and Daniela V Pachito. 2018. WHO/ILO work-related burden of disease and injury: Protocol for systematic reviews of exposure to long working hours and of the effect of exposure to long working hours on alcohol use and alcohol use disorder. Environment International,120:22-33

Hoy, D.; Brooks, P.; Woolf, A.; Blyth, F.; March, L.; Bain, C.; Baker, P.; Smith, E.; Buchbinder, R. Assessing risk of bias in prevalence studies: modification of an existing tool and evidence of interrater agreement. J Clin Epidemiol 2012;65:934-939

Lam, J.; Sutton, P.; Padula, A.M.; Cabana, M.D.; Koustas, E.; Vesterinen, H.M.; Whitaker, E.; Skalla, L.; Daniels, N.; Woodruff, T.J. Applying the Navigation Guide Systematic Review Methodology Case Study #6: Association between Formaldehyde Exposure and Asthma: A Systematic Review of the Evidence: Protocol. San Francisco, CA: University of California at San Francisco; 2016

Li, Jian, Chantal Brisson, Els Clays, Marco M Ferrario, Ivan D Ivanov, Paul Landsbergis, Nancy Leppink, Frank Pega, Hynek Pikhart, Annette M Prüss-Üstün, Reiner Rugulies, Peter L Schnall, Gretchen A Stevens, Akizumi Tsutsumi, Yuka Ujita, and Johannes Siegrist. 2018. 'WHO/ILO work-related burden of disease and injury: Protocol for systematic reviews of exposure to long working hours and of the effect of exposure to long working hours on ischaemic heart disease', Environment International, 119:558-69.

Mandrioli, Daniele, Vivi Schlünssen, Balazs Adam, Robert A Cohen, Weihong Chen, Claudio Colosio, Axel Fischer, Lode Godderis, Thomas Göen, Ivan D Ivanov, Nancy Leppink, Stefan Mandic-Rajcevic, Federica Masci, Ben Nemery, Frank Pega, Annette M Prüss-Üstün, Daria Sgargi, Yuka Ujita, Stevie Van der Mierden, Muzimkhulu Zungu, and Paul Scheepers. 2018. WHO/ILO work-related burden of disease and injury: Protocols for systematic reviews of occupational exposure to dusts and/or fibres and of the effect of occupational exposure to dusts and/or fibres on pneumoconiosis. Environment International, 119:174-185

Munn, Z.; Moola, S.; Riitano, D.; Lisy, K. The development of a critical appraisal tool for use in systematic reviews addressing questions of prevalence. Int J Health Policy Manag 2014;3:123-128

Paulo, Marillia S, Ojochide C Akagwu, Issaka Y Akparibo, Rami H Al-Rifai, Adam Balazs, Sholeh K Bazrafshan, Fabriziomaria Gobba, Ivan D Ivanov, Sanja Kezic, Nancy Leppink, Tom Loney, Alberto Modenese, Frank Pega, Cheryl Peters, Thomas D Tenkate, Yuka Ujita, Marc Wittlich, and Swen M John. Accepted. WHO/ILO work-related burden of disease and injury: Protocol for systematic reviews of occupational exposure to solar ultraviolet radiation and of the effect of occupational exposure to solar ultraviolet radiation on melanoma and non-melanoma skin cancer. Environment International.

Rooney, A.A. Protocol to evaluate the evdience for an association between perfluorooctanoic acid or perfluorooctane sulfonate exposure and immunotoxicity. in: Translation O.o.H.A.a., ed. Durham, NC: National Toxicology Program Division, National Institute of Environmental Health Sciences; 2015

Rugulies, Reiner F, Emiko Ando, Jose L Ayuso Mateos, Michela Bonafede, Maria Cabello Salmerón, Cristina Di Tecco, Dragano Nico, Quentin V Durand-Moreau, Junling Gao, Hisashi Eguchi, Ivan D Ivanov, Sergio Iavicoli, Frank Pega, Annette M Prüss-Üstün, Bruna M Rondinone, Kathrine Sørensen, Kanami Tsuno, Yuka Ujita, and Amy Zadoa. Accepted. WHO/ILO work-related burden of disease and injury: Protocol for systematic reviews of exposure to long working hours and of the effect of exposure to long working hours on depression. Environment International.

Teixeira, Liliane R, Tatiana M Azevedo, Alicja T Bortkiewicz, José U Braga, Denise T Corrêa da Silva, Wagner De Abreu, Marcia S De Almeida, Marco AN De Araújo, Elzbieta H Gadzicka, Ivan D Ivanov, Nancy Leppink, Marta RV Macedo, Elvira MGS Maciel, Małgorzata S Pawlaczyk-Łuszczyńska, Frank Pega, Annette M Prüss-Üstün, Jadwiga M Siedlecka, and Yuka Ujita. Accepted. WHO/ILO work-related burden of disease and injury: Protocol for systematic reviews of occupational exposure to noise and of the effect of occupational exposure to noise on cardiovascular disease. Environment International.

Tenkate, Thomas D, Marillia Paulo, Balazs Adam, Rami H Al-Rifai, B Ralph Chou, Fabriziomaria Gobba, Ivan D Ivanov, Nancy Leppink, Tom Loney, Frank Pega, Cheryl Peters, Annette M Prüss-Üstün, Yuka Ujita, Marc Wittlich, and Alberto Modenese. Accepted. WHO/ILO work-related burden of disease and injury: Protocol for systematic reviews of occupational exposure to solar ultraviolet radiation and of the effect of occupational exposure to solar ultraviolet radiation on cataract. Environment International.

The GATHER Working Group. The GATHER Statement: Explanation and Elaboration. Geneva: World Health Organization; 2016

Woodruff, T.J.; Sutton, P. The Navigation Guide systematic review methodology: a rigorous and transparent method for translating environmental health science into better health outcomes. Environ Health Perspect 2014;122:1007-1014

1. a Note: Limitations to GRADE’s risk of bias assessments as stated by GRADE: “First, empirical evidence supporting the criteria is limited. Attempts to show systematic difference between studies that meet and do not meet specific criteria have shown inconsistent results. Second, the relative weight one should put on the criteria remains uncertain. The GRADE approach is less comprehensive than many systems, emphasizing simplicity and parsimony over completeness. GRADE’s approach does not provide a quantitative rating of risk of bias. Although such a rating has advantages, we share with the Cochrane Collaboration methodologists a reluctance to provide a risk of bias score that, by its nature, must make questionable assumptions about the relative extent of bias associated with individual items and fails to consider the context of the individual items.” [↑](#footnote-ref-2)
2. GRADE includes a fourth type of indirectness that occurs when there are no direct (i.e., head-to-head) comparisons between two or more interventions of interest. This criterion is not relevant to our study question. [↑](#footnote-ref-3)
